# The Backscattering Problem for Time-Dependent Potentials

**DOI:** 10.1007/s00023-025-01579-7

**Published:** 2025-05-08

**Authors:** Medet Nursultanov, Lauri Oksanen, Plamen Stefanov

**Affiliations:** 1 Department of Mathematics and Statistics, University of Helsinki, Helsinki, Finland; 2 Institute of Mathematics and Mathematical Modeling, Almaty, Kazakhstan; 3 Department of Mathematics, Purdue University, West Lafayette, IN 47907 USA

**Keywords:** Primary 35P25, 35R30, Secondary 35L05, 47F05

## Abstract

We study the inverse backscattering problem for time-dependent potentials. We prove uniqueness and Lipschitz stability for the recovery of small potentials.

## Introduction

Let *q*(*t*, *x*) be smooth and supported in the cylinder $\mathbb {R}\times \Omega$, where $\Omega \subset B(0,\rho ):= \{|x|<\rho \}$ with some $\rho >0$ is a fixed domain in $\mathbb {R}^n$. We study the inverse backscattering problem for the wave equation

1 $$\begin{aligned} (\partial _t^2-\Delta +q(t,x))u=0, \quad (t,x)\in \mathbb {R}\times \mathbb {R}^n, \end{aligned}$$

$n\ge 3$, odd. We show that small enough potentials *q* are *stably* recoverable from the data.

Results for stationary potentials *q*(*x*) have been proven in [5, 6, 15, 18, 21, 27, 28]. Even though stability (say, of conditional Hölder type) has not been stated explicitly there (see also [24] for a related result), it follows from the fact that the linearization of the problem near $q=0$ is essentially the Fourier transform of *q*, see, e.g., [21]. In terms of uniqueness, the best known result is generic uniqueness so far.

The inverse problem of recovery of *q*(*t*, *x*) from “near-field” scattering data, closely related to the inverse scattering one but not restricted to backscattering, has been studied in [1, 10, 16, 19, 20, 29], and other works. Uniqueness is known, for example for potentials supported in a cylinder as above, with a tempered growth in *t*, as shown in [20]. One of the techniques is to extract the light-ray transform from the data, which relies on forward scattering, and invert it, see, e.g., [25] for an even more general situation. That transform does not see timelike singularities however, see [12, 22, 23] which makes it unstable. In view of that, the possibility of a *stable* recovery of *q* remained unclear. In [11], it was shown that a similar boundary value problem, with inputs plane waves as below, and the output measured at a fixed time $t=T$ in the whole $\mathbb {R}^n_x$, provides Lipschitz stable recovery. The proof is based on Carleman estimates.

**Fig. 1:**
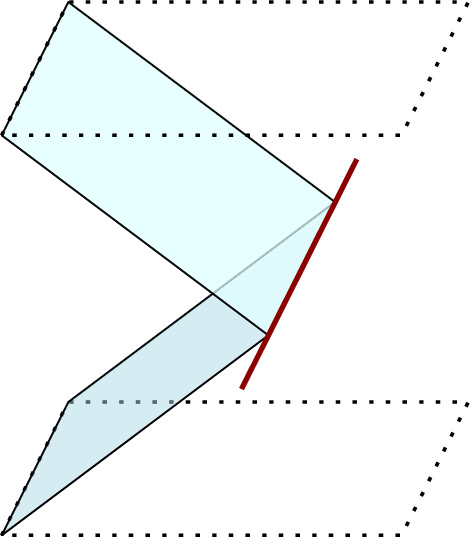
On the principal level, we integrate over the horizontal line in the middle, which is a codimension two hyperplane, actually. The support of the integrand, all terms considered, is inside the wedge, intersected with the cylinder $|x|\le \rho$

Even though forward propagating rays do not see all singularities, broken rays reflecting from the interior could, at least on the principal level. Backscattering provides such a geometry, in particular. The main reason why one can expect a stable recovery in this case is the following. Plane waves can only possibly detect singularities conormal to them, which are lightlike, indeed. On the other hand, a linearization of the backscattering data near $q=0$ is an integral over the product of one such incoming and one outgoing wave. That product, on the principal level, is supported on the intersection of such two hyperplanes in timespace, which is a delta on a codimension two (vs. one) hyperplane, see Fig. 1, where it looks like a line. That hyperplane has a richer subspace of conormals and can possibly detect non-necessarily lightlike singularities. Varying the incident direction of the incoming wave provides a complete set of conormals. We refer to the discussion in Sect. 3.3 as well.

We are restricted to small potentials, and as we pointed out already, even for stationary *q*(*x*), the uniqueness without that assumption is a well-known open problem. It seems feasible that our methods could help prove local generic uniqueness (and stability) in line with the stationary results in [5, 6, 21].

## Main Results

We describe the scattering amplitude for (1) briefly in order to formulate the main theorem. In Appendix A, we review the scattering theory for (1) in more detail.

We are sending waves $\delta (t+s-x\cdot \omega )$, where *s* is a delay parameter, $|\omega |=1$, and $t\ll 0$; let them propagate and scatter, and measure them at infinity at directions $\omega '$ and delay time $s'$. The *scattering amplitude* $A^\sharp (s',\omega ',s,\omega )$, see Definition A.2 and Proposition 1, measures the difference between the wave we sent and the scattered one. Taking $\omega ' =-\omega$, we measure the response in the direction opposite of the incoming one. If we have two potentials, $q_1$ and $q_2$, we denote the corresponding quantities by the subscripts 1 and 2.

To state our main results, we introduce the change of variables

2 $$\begin{aligned} \sigma = \frac{s - s'}{2}, \qquad \sigma ' = \frac{s + s'}{2}. \end{aligned}$$

For the choice of these coordinates we refer to Sect. 3.3. In short, we think of the data $A^\sharp (s',\omega ',s,\omega )$ as the response to the incident wave $\delta (t+s-x\cdot \omega )$, carried by the backpropagating one $\delta (t+s'+x\cdot \omega )$ (modulo more regular terms) with *s* and $s'$ chosen delay parameters. Those two waves meet at time $t=-\sigma '$ at the hyperplane $x\cdot \omega = \sigma$ in the *x*-space.

By $\tilde{A}_1^\sharp (\sigma ',\sigma ,\omega )$ and $\tilde{A}_2^\sharp (\sigma ',\sigma ,\omega )$, we denote the functions $A_1^\sharp (s',-\omega ,s,\omega )$ and $A_2^\sharp (s',-\omega ,s,\omega )$ in the new variables. Our main result is the following.

### Theorem 2.1

Let $n \ge 3$ be an odd integer, and let *q* be a smooth function supported in $\mathbb {R} \times \Omega$, where $\Omega \subset B(0, \rho )$ for some $\rho > 0$. Then, there exists $\varepsilon >0$ and $k>0$ such that if

$$\begin{aligned} \Vert q_1\Vert _{C^k(\mathbb {R}\times \bar{\Omega })}<\varepsilon , \qquad \Vert q_2\Vert _{C^k(\mathbb {R}\times \bar{\Omega })}<\varepsilon , \end{aligned}$$

then the identity $\tilde{A}_1^\sharp = \tilde{A}_2^\sharp$ implies $q_1=q_2$. Moreover, under the same assumptions on $q_1$, $q_2$, there exists a constant $C_\Omega >0$ such that

$$\begin{aligned} \Vert q_1-q_2\Vert _{L^\infty (\mathbb {R};\; L^2(\mathbb {R}^n))}\le C_\Omega \Vert \tilde{A}_1^\sharp -\tilde{A}_2^\sharp \Vert _{L^\infty (\mathbb {R}_{\sigma '}; L^2(\mathbb {S}_\omega ^{n-1}; H^{(n-1)/2}(\mathbb {R}_\sigma )))}. \end{aligned}$$

Note that we could use other norms above using complex interpolation under the assumptions of the theorem but the price for that is to make the estimate of conditional Hölder type, i.e., to have $\Vert A_1^\sharp -A_2^\sharp \Vert ^\mu$ above with some $\mu \in (0,1)$.

The restriction to odd $n\ge 3$ avoids the non-local translation representation that arises in even dimensions, see also Appendix A.

## Proofs

### A Pseudo-Linearization Identity

We review the scattering theory for time-dependent potentials in Appendix A mostly following [3, 4, 20] with some additions as well. We sketch the main notions below.

We send a plane wave $\delta (t+s-x\cdot \omega )$ to the perturbation, and let it interact with the potential. More precisely, we are solving

3 $$\begin{aligned} (\partial _t^2-\Delta +q(t,x))u^-=0, \quad u^-|_{t<-s-\rho }= \delta (t+s-x\cdot \omega ). \end{aligned}$$

Recall that $\rho$ is the radius of a cylinder which contains the support of *q*. Then, we set

4 $$\begin{aligned} u_{\text {sc}}^-= u^--\delta (t+s-x\cdot \omega ). \end{aligned}$$

The distribution $u_{\text {sc}}^-$ (which is actually a function, see Proposition 3.2) would be automatically outgoing by Definition A.1, since it vanishes for $t\ll 0$. Then, we could compute the asymptotic wave profile $u^{-,\sharp }_{\text {sc}} (s',\omega ';s,\omega )$ of $u_{\text {sc}}^- (t,x;s,\omega )$, which would give us the analog of the scattering amplitude, see Sect. A.4. As in the stationary case, we expect this to be “essentially” the kernel of the scattering operator minus identity. This is true, indeed, at least when the scattering operator exists as a bounded one as we show in Theorem A.5. One defines the scattering amplitude $A^\sharp (s',\omega ';s,\omega )$ by canceling some constant and ignoring some $s'$ derivatives.

We need to define the time-reversed analog of $u^-$ above, which we will denote by $u^\text {+} (t,x;s,\omega )$. It solves

5 $$\begin{aligned} (\partial _t^2-\Delta +q(t,x))u^\text {+} =0, \quad u^\text {+} |_{t>-s+\rho }= \delta (t+s-x\cdot \omega ). \end{aligned}$$

We want to warn the reader about a possible confusion caused by the terms incoming/outgoing. The solution *u* of (3), which we denote by $u^-$ below, is the response to an incident plane wave and it is neither incoming nor outgoing by Definition A.1. On the other hand, $u_{\text {sc}}^-=u^-_{\text {sc}}$ is outgoing. Similarly, $u^+$ is neither but $u^+_{\text {sc}}$, defined as in (4) but with *u* replaced by $u^+$, is incoming.

Let $q_1$ and $q_2$ be two such potentials, and denote the corresponding quantities with subscripts 1 and 2. We have the following formula, proven also in [26] for $n=3$, generalizing that in [21], where the potentials are time-independent.

#### Proposition 3.1

We have

6 $$\begin{aligned} (A_1^\sharp - A_2^\sharp )(s',\omega ';s,\omega ) = \int (q_1-q_2) (t ,x )u_1^-(t ,x,s,\omega )u_2^+(t,x, s',\omega ' )\, \textrm{d} t\,\textrm{d} x, \end{aligned}$$

where $u_1^-$ solves (3) with $q=q_1$, and $u_2^+$ solves (5) with $q=q_2$.

The proof of this proposition is given in Appendix A, where the necessary notations and background are introduced.

### Progressive Wave Expansion

We have the following progressive wave expansion. Let $h=:h_0$ be the Heaviside function and set $h_j (\tau ) = \tau ^j/j!$ for $\tau >0$; $h_j (\tau ) =0$ for $\tau \le 0$.

#### Proposition 3.2

([20]). Let *q* be a smooth function supported in $\mathbb {R} \times \Omega$ and $u^-$ be the solution of (3). Then, for each integer $N\ge 0$ we have

7 $$\begin{aligned} u^-(t,s,x,\omega ) = \delta (t+s-x\cdot \omega ) + \sum _{j=0}^N a_j(t,x,\omega ) h_j(t+s-x\cdot \omega )+R_N(t,x,s,\omega ) , \end{aligned}$$

where

$$\begin{aligned} a_0(t,x,\omega )&= -\frac{1}{2} \int _{-\infty }^0 q(t+\tau , x+\tau \omega )\,\textrm{d}\tau ,\\ a_j(t,x,\omega )&= -\frac{1}{2} \int _{-\infty }^0(\Box +q) a_{j-1}(t+\tau , x+\tau \omega , \omega )\, \textrm{d}\tau , \quad j=1,\dots ,N, \end{aligned}$$

and $$R_N\in C(\mathbb {R}_t\times \mathbb {R}_s\times S_\omega ^{n-1};\; H^{N+1}(\mathbb {R}^n_x))$$.

The latter statement follows from the fact that $R_N$ solves

$$\begin{aligned} (\partial _t^2-\Delta +q)R_N= - [(\partial _t^2-\Delta +q )a_N]h_N, \quad R_N|_{t<-s-\rho }=0. \end{aligned}$$

We get a similar expansion for $u^+$ but with a different remainder $R_N$, and the formulas for $a_j$ above involve integrals from 0 to $\infty$.

### Sketch of the Main Idea

Using Proposition 3.1, and keeping the most singular terms of $u_1^-$ and $u_2^+$ only, we get

8 $$\begin{aligned} \delta A^\sharp (s',\omega ';s,\omega ) \sim \int \delta q(t,x) \delta (t+s-x\cdot \omega ) \delta (t+s'-x\cdot \omega ')\,\textrm{d} t\,\textrm{d} x, \end{aligned}$$

where $\delta A^\sharp$ and $\delta q$ are formal linearizations, while the other two deltas above are Dirac deltas. The symbol $\sim$ indicates that the right-hand side captures the leading-order contribution to $\delta A^\sharp$, with possible lower-order terms omitted.

The product of the two deltas is a delta, with the coefficient $2(4-(1+\omega \cdot \omega ')^2)^{-1/2}$ on the $n-1$ dimensional hyperplane (co-dimension 2) given by the system

9 $$\begin{aligned} -t+x\cdot \omega =s, \quad -t +x\cdot \omega '=s' \end{aligned}$$

with *s*, $s'$ parameters, assuming $\omega \not =\omega '$, i.e., staying away from the forward scattering directions. Its conormal bundle is the span of $(-1,\omega )$ and $(-1,\omega ')$. Those are two lightlike covectors, and all future pointing lightlike covectors look like this. Taking linear combinations, and varying $\omega$ and $\omega '$, we get all covectors. So we are really inverting the $k=(n-1)$ – Radon transform in $\mathbb {R}^{1+n}$ (by Helgason’s terminology [7]) over *all* *k*-planes; and this is stably invertible. We must stay away from $\omega =\omega '$ though. On the other hand, the codimension two Radon transform is overdetermined, so we do not need all of them, and we can avoid the bad planes. Backscattering only ($\omega =-\omega '$) is one case where this works.

Consider the **backscattering** ($\omega '=-\omega$) problem now. Then, (9) reduces to

$$\begin{aligned} -t+x\cdot \omega =s, \quad -t -x\cdot \omega =s' \end{aligned}$$

which implies

10 $$\begin{aligned} x\cdot \omega = (s-s')/2=\sigma , \quad t = -(s+s')/2 = -\sigma '. \end{aligned}$$

This is easy to visualize as two hyperplanes in time-space at angle 45 degrees with the *t*-axis each, intersecting at a right angle, see Fig. 1.

Varying $\omega$, and $s, s'$ so that $s+s'$ is fixed, we get integrals over all “lines” (codimension two hyperplanes, actually) on the hyperplane $t=\text {const.}$, which is invertible, slice by slice. The stability estimate we get, however, treats *t* and *x* differently, in principle.

We need to analyze the lower-order terms now. If we linearize near $q\not =0$, and take the lower-order term $a_0$ for $u_1^-$ in (7) into account only, then (8) is the main term but we get the following additional terms:

$$\begin{aligned} B_1(s',\omega ';s,\omega ):= \int \delta q(t,x) a_1(t,x,\omega ) h(t+s-x\cdot \omega ) \delta (t+s'-x\cdot \omega ')\,\textrm{d} t\,\textrm{d} x, \end{aligned}$$

plus a similar $B_2$ term coming from $u_1^+$, plus an even more regular term containing two Heaviside functions *h*. Each of $B_1$, $B_2$ integrates $\delta q$ over a (weighted) truncated delta on the hyperplane $t+s'-x\cdot \omega '=0$, or $t+s-x\cdot \omega =0$, respectively, see Fig. 1. In other words, the Schwartz kernels are deltas on half-hyperplanes. They can be thought of as a superposition of deltas on codimension two hyperplanes, as in (8), all parallel to the intersection in Fig. 1, moving along the corresponding wing of the wedge there. We just need upper bounds of $B_1$, $B_2$, and they can be done in the same norms as those we use for (8) to get an $O(\varepsilon \Vert q\Vert )$ perturbation. The $\varepsilon$ gain comes from $a_1$. We can treat the more regular terms, coming from $a_j$, $j\ge 1$, and from $R_N$ in Proposition 3.2 similarly, which is a tedious task but doable. An important observation is that the support of the product $u_1^- u_2^+$ in (6) is contained in the intersection of the half-space above the lower hyperplane (due to $u_1^-$, see (43)) and the half-space below the upper hyperplane (due to $u_1^+$), i.e., on the left of that the wedge in the figure. This is further intersected with the cylinder $|x|\le \rho$, so in particular, we integrate in (6) over a compact set depending on the parameters.

### Proof of the Main Result

Here, we will prove our main result. We begin with a few notations. Inspired by (6), we set

$$\begin{aligned} q(t,x) = q_1(t,x) - q_2(t,x) \end{aligned}$$

and

$$\begin{aligned} Mq (s', s,\omega ) = \int q(t ,x )u_1^-(t ,x,s,\omega )u_2^+(t,x,s',-\omega )\, \textrm{d} t\,\textrm{d} x. \end{aligned}$$

Writing

$$\begin{aligned} u_1^- (t ,x,s,\omega )&= \delta (t+s- x\cdot \omega )+u_{1,\mathrm sc}^-(t ,x,s,\omega )\\ u_2^+(t ,x,s,-\omega )&= \delta (t+s+ x\cdot \omega )+u_{2,\mathrm sc}^+(t ,x,s,-\omega ) \end{aligned}$$

we get

$$\begin{aligned} M = M_{00}+M_{01}+ M_{10}+ M_{11}, \end{aligned}$$

where

11 $$\begin{aligned} M_{00}q(s',s,\omega )&= \int q(t,x) \delta (t+s-x\cdot \omega ) \delta (t+s'+x\cdot \omega )\,\textrm{d} t\,\textrm{d} x, \nonumber \\ M_{10}q(s',s,\omega )&= \int q(t,x) u_{1,\mathrm sc}^- (t,x,s,\omega ) \delta (t+s'+x\cdot \omega )\,\textrm{d} t\,\textrm{d} x,\nonumber \\ M_{01}q(s',s,\omega )&= \int q(t,x) \delta (t+s-x\cdot \omega ) u_{2,\mathrm sc}^+ (t, x,s', -\omega )\,\textrm{d} t\,\textrm{d} x,\nonumber \\ M_{11}q(s',s,\omega )&= \int q(t,x) u_{1,\mathrm sc}^-(t,x,s,\omega ) u_{2,\mathrm sc}^+(t,x, s', -\omega )\,\textrm{d} t\,\textrm{d} x. \end{aligned}$$

We denote by $\tilde{M}q$, $\tilde{M}_{kl}q$ the above functions in the variables given by (2).

#### Remark 3.1

Let

12 $$\begin{aligned} \Sigma = [-\rho , \rho ], \end{aligned}$$

where $\rho$ is the radius of a cylinder which contains the support of *q*. Note that if $\sigma \notin \Sigma$, then (10) does not hold for any $x\in \Omega$ and $\omega \in \mathbb {S}^{n-1}$, and hence, the line of intersection (a hyperplane) in Fig. 1 does not intersect the cylinder $\mathbb {R}\times \Omega$. Therefore, $\tilde{M}_{00}q(\sigma ',\sigma ,\omega )= 0$ for $\sigma \notin \Sigma$. If $\sigma \in \Sigma$, due to the definitions of $u_{1,sc}^+$ and $u_{2,sc}^+$, it follows that there exists $T>0$ such that the (*t*, *x*)-supports of the integrands in the definition of $\tilde{M}q$, $\tilde{M}_{kl}q$ belong to $(-T - \sigma ',T - \sigma )\times \Omega$.

Next, we will study $\tilde{M}_{kl}q$. We begin with $\tilde{M}_{00}q$:

#### Lemma 3.1

Let $\Sigma = [-\rho , \rho ]$. There exists a constant $C>0$ depending only on *n* such that

13 $$\begin{aligned} \Vert q\Vert _{L^\infty (\mathbb {R};\; L^2(\mathbb {R}^n))} / C\le \Vert \tilde{M}_{00}q\Vert _{L^\infty (\mathbb {R}_{\sigma '}; L^2(\mathbb {S}_\omega ^{n-1}; H^{(n-1)/2}(\Sigma _\sigma )))} \le C \Vert q\Vert _{L^\infty (\mathbb {R};\; L^2(\mathbb {R}^n))}. \end{aligned}$$

#### Proof

As explained in Sect. 3.3, the product of the deltas in (11) can also be written as

$$\begin{aligned} \delta \left( t+ \frac{s+s'}{2}\right) \delta \left( \frac{s-s'}{2} - x\cdot \omega \right) . \end{aligned}$$

Then,

$$\begin{aligned} M_{00}q(s',s,\omega ) = \left[ Rq(-(s'+s)/2, \cdot )\right] ((s-s')/2,\omega ), \end{aligned}$$

where $Rf(p,\omega )= \int \delta (p-x\cdot \omega ) f(x)\,\textrm{d} x$ is the Radon transform of *f*. Using the change of variables given by (2), we rewrite

$$\begin{aligned} \tilde{M}_{00}q(\sigma ',\sigma ,\omega ) = \left[ Rq(-\sigma ', \cdot )\right] (\sigma ,\omega ). \end{aligned}$$

For every *s*, we have the following stability estimate for the Radon transform, see [14, Ch. 2]:

$$\begin{aligned} \Vert f\Vert _{H^s(\mathbb {R}^n)}/C \le \Vert Rf\Vert _{L^2(\mathbb {S}_\omega ^{n-1}; H^{s + (n-1)/2}(\mathbb {R}_\sigma ))} \le C\Vert f\Vert _{H^s(\mathbb {R}^n)}, \end{aligned}$$

for some constant $C>0$. Therefore, choosing $s=0$, we obtain

$$\begin{aligned} \Vert q(-\sigma ',\cdot )\Vert _{L^2(\mathbb {R}^n)}/C \le \Vert \tilde{M}_{00} q(\sigma ',\cdot ,\cdot )\Vert _{L^2(\mathbb {S}_\omega ^{n-1}; H^{(n-1)/2}(\mathbb {R}_\sigma ))} \le C\Vert q(-\sigma ',\cdot )\Vert _{L^2(\mathbb {R}^n)} \end{aligned}$$

for every $\sigma '$ with *C* independent of it. Finally, as we noted in Remark 3.1,

$$\begin{aligned} \Vert \tilde{M}_{00}q\Vert _{L^\infty (\mathbb {R}_{\sigma '}; L^2(\mathbb {S}_\omega ^{n-1}; H^{(n-1)/2}(\Sigma _\sigma )))} = \Vert \tilde{M}_{00}q\Vert _{L^\infty (\mathbb {R}_{\sigma '}; L^2(\mathbb {S}_\omega ^{n-1}; H^{(n-1)/2}(\mathbb {R}_\sigma )))}, \end{aligned}$$

and hence, (13) holds. $\square$

We need the next lemmas to obtain similar results for $\tilde{M}_{10}q$ and $\tilde{M}_{01}q$.

#### Lemma 3.2

Let $\Sigma$ and *T* be as in Remark 3.1. Let $a_{1,j}$, $h_j$, and $R_{1,N}$ be the functions introduced in Sect. 3.2 with $q=q_1$ and let $\bar{R}_{1,N}$ be the function such that

$$\begin{aligned} \bar{R}_{1,N} (t,x, t + s - x\cdot \omega ,\omega ) = R_{1,N}(t,x,s,\omega ). \end{aligned}$$

Then, there exists $C_{\Omega ,N}>0$ such that

$$\begin{aligned}&\Vert \tilde{M}_{10}q\Vert _{L^\infty (\mathbb {R}_{\sigma '};L^2(\mathbb {S}_\omega ^{n-1}; H^{(n-1)/2}(\Sigma _\sigma )))} \le C_{\Omega ,N} \Vert q\Vert _{L^\infty (\mathbb {R}; L^2(\mathbb {R}^n))}\\  &\quad \times \left( \sum _{j=0}^N \Vert a_{1,j} \Vert _{L^\infty (\mathbb {R}\times \mathbb {S}^{n-1};C^{2n-2}(\bar{\Omega }))} \right. \\  &\qquad \left. + \sup _{\sigma '\in \mathbb {R}}\int _{-T-\sigma '}^{T-\sigma '} \sup _{\omega \in \mathbb {S}^{n-1}}\Vert \bar{R}_{1,N} (t,\cdot , 2t + 2\sigma ',\omega )\Vert _{C^{2n-2}(\bar{\Omega })}\textrm{d}t\right) . \end{aligned}$$

#### Proof

For the sake of brevity, we denote

$$\begin{aligned} U(t,x, t + s - x\cdot \omega ,\omega ) = \sum _{j=0}^N a_{1,j}(t,x,\omega ) h_j(t+s-x\cdot \omega ) + \bar{R}_{1,N} (t,x, t + s - x\cdot \omega ,\omega ). \end{aligned}$$

Then,

$$\begin{aligned} M_{10}q(s',s,\omega )&= \int _{\mathbb {R}^n} \int _{\mathbb {R}} q(t,x) U (t,x, t + s - x\cdot \omega ,\omega ) \delta (t+s'+x\cdot \omega )\,\textrm{d} t\,\textrm{d} x\\&= \int _{\mathbb {R}} \int _{\omega ^\perp } q(t,-(t + s') \omega + y) U(t,-(t + s') \omega + y, 2t + s + s',\omega ) \,\textrm{d} t\,\textrm{d} y. \end{aligned}$$

Using the change of variables given by (2), for any $\sigma \in \Sigma$, we obtain

$$\begin{aligned} \tilde{M}_{10}q(\sigma ',\sigma ,\omega )&= \int _{-T - \sigma '}^{T - \sigma '} \int _{\omega ^\perp } q(t,-(t + \sigma ' - \sigma ) \omega + y) U(t,-(t + \sigma ' - \sigma ) \omega \\&\quad + y, 2t + 2\sigma ',\omega ) \,\textrm{d} t\,\textrm{d} y\\&=\int _{-T - \sigma '}^{T - \sigma '} \left[ R_{U(t,\cdot ,2t + 2\sigma ',\cdot )}q(t,\cdot )\right] (-t + \sigma - \sigma ',\omega )\,\textrm{d} t, \end{aligned}$$

where

$$\begin{aligned} R_\mu f (p,\omega ) = \int _{x\cdot \omega =p} \mu (x,\omega ) f(x) \textrm{d}x \end{aligned}$$

is the weighted Radon transform of *f* with weight $\mu$. By Theorem B.1,

$$\begin{aligned}&\Vert \tilde{M}_{10}q(\sigma ',\cdot ,\cdot )\Vert _{L^2(\mathbb {S}_\omega ^{n-1}; H^{(n-1)/2}(\Sigma _\sigma ))}\\  &\quad \le C_\Omega \sup _{t\in [-T - \sigma ',T-\sigma ']}\Vert q(t,\cdot )\Vert _{L^2(\Omega )} \int _{-T-\sigma '}^{T-\sigma '} \sup _{\omega \in \mathbb {S}^{n-1}}\Vert U (t,\cdot , 2t + 2\sigma ',\omega )\Vert _{C^{2n-2}(\bar{\Omega })}\text {d} t.\end{aligned}$$

We estimate next

$$\begin{aligned}&\int _{-T-\sigma '}^{T-\sigma '} \sup _{\omega \in \mathbb {S}^{n-1}}\Vert U (t,\cdot , 2t + 2\sigma ',\omega )\Vert _{C^{2n-2}(\bar{\Omega })}\text {d} t\\  &\qquad \le C_N \int _{-T-\sigma '}^{T-\sigma '} \sup _{\omega \in \mathbb {S}^{n-1}}\Vert a (t,\cdot ,\omega )\Vert _{C^{2n-2}(\bar{\Omega })}\text {d} t \\  &\qquad \quad + \int _{-T-\sigma '}^{T-\sigma '} \sup _{\omega \in \mathbb {S}^{n-1}}\Vert \bar{R}_{1,N} (t,\cdot , 2t + 2\sigma ',\omega )\Vert _{C^{2n-2}(\bar{\Omega })}\text {d} t. \end{aligned}$$

The last two estimates complete the proof. $\square$

#### Lemma 3.3

Let $\Omega \subset \mathbb {R}^n$ be a bounded set, $\alpha$ be a multi-index, and $K = |\alpha | +(n+1)/2$. Let $f\in C^N(\mathbb {R}_t\times \mathbb {R}_s\times \mathbb {R}^n_x)$ such that $f(t,s,\cdot )$ has a compact support. Assume that $N\in \mathbb {N}$ is large enough so that the equation

$$\begin{aligned} {\left\{ \begin{array}{ll} (\partial _t^2 - \Delta + q(t,x))v(t,s,x) = f(t,s,x),\\ v\arrowvert _{t<-s-\rho } = 0. \end{array}\right. } \end{aligned}$$

has the unique smooth (as much as needed) solution *v*. Then,

14 $$\begin{aligned} \Vert D_x^\alpha v\Vert _{L^\infty (\Omega )} \le C_{\Omega ,K} e^{C_q^K(t+s+\rho )} \int _{-s-\rho }^t \Vert f(\tau ,s,\cdot )\Vert _{H^K(\mathbb {R}^n)}\textrm{d}\tau , \end{aligned}$$

where

$$\begin{aligned} C_q^K = 2 + C_K\Vert q(t,\cdot )\Vert _{C^k(\mathbb {R}^n)} \end{aligned}$$

with some $C_K>0$ dependent on *K*.

#### Proof

For non-negative $k\in \mathbb {Z}$, we define

$$\begin{aligned} E_s^k(t) = \Vert \partial _tv(t,s,\cdot )\Vert _{H^k(\mathbb {R}^n)}^2 + \sum _{|\gamma |\le k} \Vert \nabla _x D_x^\gamma v(t,s,\cdot )\Vert _{(L^2(\mathbb {R}^n))^n}^2 + \Vert v(t,s,\cdot )\Vert _{L^2(\mathbb {R}^n)}^2. \end{aligned}$$

Then,

$$\begin{aligned} \partial _t E_s^k(t)= &   2 \sum _{|\gamma |\le k} \Re (\partial _t^2 D_x^\gamma v(t,s,\cdot ), \partial _t D_x^\gamma v(t,s,\cdot ))_{L^2(\mathbb {R}^n)} \\  &   - 2 \sum _{|\gamma |\le k} \Re (\Delta D_x^\gamma v(t,s,\cdot ), \partial _t D_x^\gamma v(t,s,\cdot ) )_{L^2(\mathbb {R}^n)}\\    &   + 2\Re (\partial _t v(t,s,\cdot ), v(t,s,\cdot ))_{L^2(\mathbb {R}^n)}, \end{aligned}$$

where $\Re$ denotes the real part of the number. Since

$$\begin{aligned} (\partial _t^2 - \Delta )D_x^\gamma v(t,s,x) + D_x^\gamma (q(t,x)v(t,s,x)) = D_x^\gamma f(t,s,x), \end{aligned}$$

the last identity becomes

$$\begin{aligned} \partial _t E_s^k(t)= &   2 \sum _{|\gamma |\le k} \Re (D_x^\gamma f(t,s,\cdot ), \partial _t D_x^\gamma v(t,s,\cdot ))_{L^2(\mathbb {R}^n)} \\  &   - 2 \sum _{|\gamma |\le k}\Re (D_x^\gamma (q(t,\cdot ) v(t,s,\cdot )), \partial _tD_x^\gamma v(t,s,\cdot ) )_{L^2(\mathbb {R}^n)} \\  &   + 2\Re (\partial _t v(t,s,\cdot ), v(t,s,\cdot ))_{L^2(\mathbb {R}^n)}. \end{aligned}$$

Using the Cauchy–Schwarz inequality, we obtain

$$\begin{aligned} \partial _t E_s^k(t) \le \sum _{|\gamma |\le k} \Vert D_x^\gamma f(t,s,\cdot )\Vert _{L^2(\mathbb {R}^n)}^2 + (2 + C_k\Vert q(t,\cdot )\Vert _{C^k(\mathbb {R}^n)} ) E_s^k(t). \end{aligned}$$

By integration over $(-s-\rho , t)$, we obtain

$$\begin{aligned} E_s^k(t) \le F_s^k(t) + C_q^k \int _{-s-\rho }^t E_s^k(\tau ) \text {d}\tau , \end{aligned}$$

where

$$\begin{aligned} F_s^k(t) = \int _{-s-\rho }^t \Vert f(\tau ,s,\cdot )\Vert _{H^k(\mathbb {R}^n)}\text {d}\tau , \qquad C_q^k = 2 + C_k\Vert q(t,\cdot )\Vert _{C^k(\mathbb {R}^n)}. \end{aligned}$$

Then, the Grönwall’s inequality implies

$$\begin{aligned} E_s^k(t) \le F_s^k(t) + C_q^k \int _{-s-\rho }^t F_s^k(\tau ) e^{C_q^k(t - \tau )}\text {d}\tau . \end{aligned}$$

Since $F_s^k$ is an increasing function,

15 $$\begin{aligned} E_s^k(t) \le F_s^k(t)\left( 1 + C_q^k \int _{-s-\rho }^t e^{C_q^k(t - \tau )}\text {d}\tau \right) \le F_s^k(t) e^{C_q^k(t+s+\rho )}. \end{aligned}$$

Due to the Sobolev inequality, it follows that

$$\begin{aligned} \Vert v(t,s,\cdot )\Vert _{C^{|\alpha |}(\bar{\Omega })} \le C_{\Omega ,K} \Vert v(t,s,\cdot )\Vert _{H^{K+1}(\Omega )} \le C_{\Omega ,K} \Vert v(t,s,\cdot )\Vert _{H^{K+1}(\mathbb {R}^n)}. \end{aligned}$$

Combining this with (15), we obtain (14). $\square$

#### Lemma 3.4

Let $L = 2n - 2$. There exists a sufficiently large $N\in \mathbb {R}$ such that if

16 $$\begin{aligned} \Vert q_1\Vert _{C^{L + \frac{n-1}{2}+3+2N}(\mathbb {R}\times \bar{\Omega })}<1, \end{aligned}$$

then,

$$\begin{aligned} \sum _{j=0}^N \Vert a_{1,j} \Vert _{L^\infty (\mathbb {R}\times \mathbb {S}^{n-1};C^{2n-2}(\bar{\Omega }))} \le C_{\Omega ,N} \Vert q_1\Vert _{C^{L + 2N}(\mathbb {R}\times \bar{\Omega })} \end{aligned}$$

and

$$\begin{aligned} \sup _{\sigma '\in \mathbb {R}}\int _{-T-\sigma '}^{T-\sigma '} \sup _{\omega \in \mathbb {S}^{n-1}}\Vert \bar{R}_{1,N} (t,\cdot , 2t + 2\sigma ',\omega )\Vert _{C^{L} (\bar{\Omega })}\textrm{d} t \le C_{\Omega ,N} \Vert q_1\Vert _{C^{L + \frac{n-1}{2}+3+2N}(\mathbb {R}\times \bar{\Omega })}. \end{aligned}$$

#### Proof

The first estimate comes directly from the definition of $a_{1,j}$. It remains to show the second estimate. Set

$$\begin{aligned} A = \sup _{\sigma '\in \mathbb {R}}\int _{-T-\sigma '}^{T-\sigma '} \sup _{\omega \in \mathbb {S}^{n-1}}\Vert \bar{R}_{1,N} (t,\cdot , 2t + 2\sigma ',\omega )\Vert _{C^{L}(\bar{\Omega })}\text {d} t. \end{aligned}$$

We write

$$\begin{aligned} A&= \sup _{\sigma '\in \mathbb {R}}\int _{-T}^{T} \sup _{\omega \in \mathbb {S}^{n-1}}\Vert \bar{R}_{1,N} (t-\sigma ',\cdot , 2t ,\omega )\Vert _{C^{L}(\bar{\Omega })}\text {d} t \\  &= \sup _{\sigma '\in \mathbb {R}}\int _{-T}^{T} \sup _{\omega \in \mathbb {S}^{n-1}} \sum _{|\gamma |\le L}\sup _{x\in \Omega } \left| [D_x^\gamma \bar{R}_{1,N}](t-\sigma ',x, 2t,\omega )\right| \text {d}t\\  &= \sup _{\sigma '\in \mathbb {R}}\int _{-T}^{T} \sup _{\omega \in \mathbb {S}^{n-1}} \sum _{|\alpha |+|\beta |\le L}\sup _{x\in \Omega } \left| [D_x^{\alpha }D_{s}^{|\beta |}R_{1,N}](t-\sigma ',x, 2t - (t-\sigma ') + x\cdot \omega ,\omega )\right| \text {d}t. \end{aligned}$$

Then, we estimate

17 $$\begin{aligned} A&\le \sum _{|\alpha |+|\beta |\le L}2T\sup _{\sigma '\in \mathbb {R}} \sup _{\omega \in \mathbb {S}^{n-1}} \sup _{x\in \Omega } \sup _{t\in [-T,T]} \left| [D_x^{\alpha }D_{s}^{|\beta |}R_{1,N}](t-\sigma ',x, 2t - (t-\sigma ') + x\cdot \omega ,\omega )\right| \nonumber \\&\le \sum _{|\alpha |+|\beta |\le L}2T\sup _{t\in \mathbb {R}} \sup _{\omega \in \mathbb {S}^{n-1}} \sup _{x\in \Omega } \sup _{s\in [-2T,2T]} \left| [D_x^{\alpha }D_{s}^{|\beta |}R_{1,N}](t,x, s - t + x\cdot \omega ,\omega )\right| . \end{aligned}$$

To estimate the last term, let us fix multi-indexes $\alpha$, $\beta$ such that $|\alpha | + |\beta | = L$ and note that

$$\begin{aligned} (\partial _t^2-\Delta +q)D_{s}^{|\beta |}R_{1,N}(t,x,s,\omega )= - [(\partial _t^2-\Delta +q_1 )a_{1,N}](t,x,\omega )h_{N}^{(|\beta |)}(s + t - x\cdot \omega ). \end{aligned}$$

Moreover, by employing the derivative definition, it can be verified that

$$\begin{aligned} D_{s}^{|\beta |}R_{1,N}(t,x,s,\omega ) \arrowvert _{t < -s - \rho } = 0. \end{aligned}$$

We denote

18 $$\begin{aligned} A_{1,N}(\tau ,x,\omega ) = -[(\partial _t^2-\Delta +q_1)a_{1,N}](t,x,\omega ), \end{aligned}$$

Next, we will show that

19 $$\begin{aligned} f(t,s,x,\omega ) = A_{1,N}(\tau ,x,\omega )h_{N}^{(|\beta |)}(s + t - x\cdot \omega ) \end{aligned}$$

has a compact support as a function of the *x* variable. In [20], it was shown that

$$\begin{aligned} a_{1,N}(t,x,\omega ) = 0, \quad \text {for } x\cdot \omega <-\rho \text { and for } |x - x\cdot \omega |>\rho . \end{aligned}$$

Hence, if $x\cdot \omega \le 0$, for sufficiently large |*x*|, it follows that $a_{1,N}(t,x,\omega ) = 0$. If $x\cdot \omega > 0$, then $h_{N}^{(|\beta |)}(s + t - x\cdot \omega ) = 0$ for sufficiently large |*x*|. Therefore, for fixed *t*, *s*, and $\omega$, the function given by (19) is compactly supported. Therefore, by Lemma 3.3,

$$\begin{aligned}&\sup _{x\in \Omega }|D^\alpha _xD_{s}^{|\beta |}R_{1,N}(t,x,s,\omega ) |\\  &\quad \le C_{\Omega ,K} e^{C_q^K(t+s+\rho )} \int _{-s-\rho }^t \Vert A_{1,N}(\tau ,\cdot ,\omega )h_{N}^{(|\beta |)}(s + \tau - (\cdot )\cdot \omega )\Vert _{H^K(\mathbb {R}^n)}\text {d}\tau , \end{aligned}$$

where

20 $$\begin{aligned} K = |\alpha | + \frac{n-1}{2} +1, \qquad C_q^K = 2 + C_K\Vert q(t,\cdot )\Vert _{C^K(\mathbb {R}^n)}. \end{aligned}$$

Since $h_{N}^{(|\beta |+k)}$ is an increasing function for all $k=0,\cdots ,K$, it follows that

$$\begin{aligned}  &   \sup _{x\in \Omega }|D^\alpha _xD_{s}^{|\beta |}R_{1,N}(t,x,s,\omega ) | \le C_{\Omega ,K} e^{C_q^K(t+s+\rho )} (t + s +\rho )\\  &   \quad \times \sup _{\tau \in \mathbb {R}}\Vert A_{1,N}(\tau ,\cdot ,\omega )h_{N}^{(|\beta |)}(s + t - (\cdot )\cdot \omega )\Vert _{H^K(\mathbb {R}^n)}. \end{aligned}$$

This is true for all $s\in \mathbb {R}$. Hence, if we choose $y\in \Omega$ and replace *s* by $s - t +y\cdot \omega$, the last estimate becomes

$$\begin{aligned}  &   \sup _{x\in \Omega }|[D^\alpha _xD_{s}^{|\beta |}R_{1,N}](t,x,s - t +y\cdot \omega ,\omega ) | \le C_{\Omega ,K} e^{C_q^K(s+y\cdot \omega +\rho )} (s+y\cdot \omega +\rho )\\  &   \quad \times \sup _{\tau \in \mathbb {R}}\Vert A_{1,N}(\tau ,\cdot ,\omega )h_{N}^{(|\beta |)}(s + y\cdot \omega - (\cdot )\cdot \omega )\Vert _{H^K(\mathbb {R}^n)}. \end{aligned}$$

Let

$$\begin{aligned} z = \sup _{s\in [-2T,2T]} \sup _{y\in \Omega } \sup _{\omega \in \mathbb {S}^{n-1}} (s + y\cdot \omega ). \end{aligned}$$

Note that *z* is constant depending only on $\Omega$. Then, from the last inequality, we derive

21 $$\begin{aligned}  &   \sup _{x,y\in \Omega }\sup _{t\in \mathbb {R}}\sup _{s\in [-2T,2T]}|[D^\alpha _xD_{s}^{|\beta |}R_{1,N}](t,x,s - t +y\cdot \omega ,\omega ) | \le C_{\Omega ,K} e^{C_q^K(z+\rho )} (z+\rho )\nonumber \\  &   \quad \times \sup _{\tau \in \mathbb {R}}\Vert A_{1,N}(\tau ,\cdot ,\omega )h_{N}^{(|\beta |)}(z - (\cdot )\cdot \omega )\Vert _{H^K(\mathbb {R}^n)}. \end{aligned}$$

Moreover, we know that

$$\begin{aligned} A_{1,N}(\tau ,\cdot ,\omega )h_{N}^{(|\beta |)}(z - (\cdot )\cdot \omega ) \end{aligned}$$

has a compact support with respect to *x*, which is uniformly bounded in $\tau \in \mathbb {R}$ and $\omega \in \mathbb {S}^{n-1}$, that is, there is a compact set $\tilde{\Omega }$ such that

$$\begin{aligned} {{\,\textrm{supp}\,}}A_{1,N}(\tau ,\cdot ,\omega )h_{N}^{(|\beta |)}(z - (\cdot )\cdot \omega ) \subset \tilde{\Omega } \qquad \text {for all } \tau \in \mathbb {R} \text { and }\omega \in \mathbb {S}^{n-1}. \end{aligned}$$

The set $\tilde{\Omega }$ depends only on $\Omega$. Therefore,

22 $$\begin{aligned}  &   \sup _{\tau \in \mathbb {R}}\Vert A_{1,N}(\tau ,\cdot ,\omega )h_{N}^{(|\beta |)}(z - (\cdot )\cdot \omega )\Vert _{H^K(\mathbb {R}^n)} \nonumber \\    &   \quad \le \sup _{\tau \in \mathbb {R}}\Vert A_{1,N}(\tau ,\cdot ,\omega )h_{N}^{(|\beta |)}(z - (\cdot )\cdot \omega )\Vert _{H^K(\tilde{\Omega })} \nonumber \\  &   \quad \le C_{\Omega ,N} \sup _{\tau \in \mathbb {R}}\Vert A_{1,N}(\tau ,\cdot ,\omega )\Vert _{H^K(\tilde{\Omega })}. \end{aligned}$$

Therefore, since

$$\begin{aligned}  &   \sup _{x\in \Omega }\sup _{t\in \mathbb {R}}\sup _{s\in [-2T,2T]}|[D^\alpha _xD_{s}^{|\beta |}R_{1,N}](t,x,s - t +x\cdot \omega ,\omega ) |\\  &   \quad \le \sup _{x,y\in \Omega }\sup _{t\in \mathbb {R}}\sup _{s\in [-2T,2T]}|[D^\alpha _xD_{s}^{|\beta |}R_{1,N}](t,x,s - t +y\cdot \omega ,\omega ) |, \end{aligned}$$

from (21), (20), and (16), we obtain

$$\begin{aligned}  &   \sup _{x\in \Omega }\sup _{t\in \mathbb {R}}\sup _{s\in [-2T,2T]}\sup _{\omega \in \mathbb {S}^{n-1}}|[D^\alpha _xD_{s}^{|\beta |}R_{1,N}](t,x,s - t +x\cdot \omega ,\omega ) |\\  &   \quad \le C_{\Omega ,N} \sup _{\omega \in \mathbb {S}^{n-1}}\sup _{\tau \in \mathbb {R}}\Vert A_{1,N}(\tau ,\cdot ,\omega )\Vert _{C^{K}(\tilde{\Omega })}. \end{aligned}$$

Since

23 $$\begin{aligned} \sup _{\omega \in \mathbb {S}^{n-1}}\sup _{\tau \in \mathbb {R}}\Vert A_{1,N}(\tau ,\cdot ,\omega )\Vert _{C^K(\tilde{\Omega })} \le C_{\Omega ,N} \Vert q_1\Vert _{C^{K+2+2N}(\mathbb {R}\times \bar{\Omega })}, \end{aligned}$$

the previous estimate implies

$$\begin{aligned}  &   \sup _{x\in \Omega }\sup _{t\in \mathbb {R}}\sup _{s\in [-2T,2T]}\sup _{\omega \in \mathbb {S}^{n-1}}|[D^\alpha _xD_{s}^{|\beta |}R_{1,N}](t,x,s - t +x\cdot \omega ,\omega ) | \\    &   \quad \le C_{\Omega ,N} \Vert q_1\Vert _{C^{K+2+2N}(\mathbb {R}\times \bar{\Omega })}. \end{aligned}$$

Then, from (17), it follows that

$$\begin{aligned} A \le C_{\Omega ,N} \Vert q_1\Vert _{C^{L + \frac{n-1}{2}+3+2N}(\mathbb {R}\times \bar{\Omega })}. \end{aligned}$$

This completes the proof. $\square$

Now, we are ready to estimate $\tilde{M}_{10}q$. Similarly, the same holds for $\tilde{M}_{01}q$.

#### Lemma 3.5

Let $\Sigma$ be as in (12). There exists a sufficiently large $k\in \mathbb {N}$ such that if

24 $$\begin{aligned} \Vert q_1\Vert _{C^{k}(\mathbb {R}\times \bar{\Omega })} \le \varepsilon < 1, \end{aligned}$$

then

25 $$\begin{aligned} \Vert \tilde{M}_{10}q\Vert _{L^\infty (\mathbb {R}_{\sigma '};L^2(\mathbb {S}_\omega ^{n-1}; H^{(n-1)/2}(\Sigma _\sigma )))} \le \varepsilon C_{\Omega } \Vert q\Vert _{L^\infty (\mathbb {R}; L^2(\mathbb {R}^n))}. \end{aligned}$$

#### Proof

Depending on $\Omega$, we choose $N\in \mathbb {N}$ large enough so that the hypothesis of Lemma 3.4 is satisfied. Let $k=L + \frac{n-1}{2}+3+2N$. Then, Lemmas 3.2, 3.4, and (24) give (25). $\square$

Due to the symmetry, the same estimate holds for $\tilde{M}_{01}q$. Next, we obtain a similar result for $M_{11}q$.

#### Lemma 3.6

Let $\Sigma$ be as in (12). There exists a sufficiently large $k\in \mathbb {R}$ such that if

$$\begin{aligned} \Vert q_1\Vert _{C^{k}(\mathbb {R}\times \bar{\Omega })} \le \varepsilon< 1, \qquad \Vert q_2\Vert _{C^{k}(\mathbb {R}\times \bar{\Omega })} \le \varepsilon < 1, \end{aligned}$$

then

26 $$\begin{aligned} \Vert \tilde{M}_{11}q\Vert _{L^\infty (\mathbb {R}_{\sigma '};L^2(\mathbb {S}_\omega ^{n-1}; H^{(n-1)/2}(\Sigma _\sigma )))} \le \varepsilon C_{\Omega } \Vert q\Vert _{L^\infty (\mathbb {R}; L^2(\mathbb {R}^n))}. \end{aligned}$$

#### Proof

Let $N\in \mathbb {N}$ be sufficiently large. We set

$$\begin{aligned}&Q_j^k(t,x,\omega ) = q(t,x) a_{1,j}(t,x,\omega )a_{2,k}(t,x,-\omega ),\\&Q_j(t,x,\omega ) = q(t,x) a_{1,j}(t,x,\omega ),\\&Q^k(t,x,\omega ) = q(t,x) a_{2,k}(t,x,-\omega ). \end{aligned}$$

Next, we define

$$\begin{aligned}&A_{kj}^l(\sigma ',\sigma ,\omega ) = \int _{\Omega }\int _{-T-\sigma '}^{T - \sigma '}Q_j^k(t,x,\omega ) h_j^{(l)}(t + \sigma ' + \sigma - x\cdot \omega ) \delta (t + \sigma ' - \sigma + x\cdot \omega ) \textrm{d}t\textrm{d}x, \\&B_{kj}^l(\sigma ',\sigma ,\omega ) = \int _{\Omega }\int _{-T-\sigma '}^{T - \sigma '}Q_j^k(t,x,\omega ) \delta (t + \sigma ' + \sigma - x\cdot \omega ) h_k^{(l)}(t + \sigma ' - \sigma + x\cdot \omega ) \textrm{d}t\textrm{d}x, \\&A_k^l(\sigma ',\sigma ,\omega ) = \int _{\Omega }\int _{-T-\sigma '}^{T - \sigma '}Q^k(t,x,\omega ) \partial _{\sigma }^{l}R_{1,N}(t,x,\sigma ' + \sigma ,\omega ) \delta (t + \sigma ' - \sigma + x\cdot \omega ) \textrm{d}t\textrm{d}x,\\&B_j^l(\sigma ',\sigma ,\omega ) = \int _{\Omega }\int _{-T-\sigma '}^{T - \sigma '}Q_j(t,x,\omega ) \delta (t + \sigma ' + \sigma - x\cdot \omega ) \partial _{\sigma }^{l} R_{2,N}(t,x,\sigma '-\sigma ,\omega ) \textrm{d}t\textrm{d}x \end{aligned}$$

and

$$\begin{aligned}&A_j^{l_1l_2}(\sigma ',\sigma ,\omega ) = \int _{\Omega }\int _{-T-\sigma '}^{T - \sigma '}Q_j(t,x,\omega ) h_j^{(l_1)}(t + \sigma ' + \sigma - x\cdot \omega ) \partial _{\sigma }^{l_2} R_{2,N}(t,x,\sigma '-\sigma ,\omega ) \textrm{d}t\textrm{d}x, \\&B_{k}^{l_1l_2}(\sigma ',\sigma ,\omega ) = \int _{\Omega }\int _{-T-\sigma '}^{T - \sigma '}Q^k(t,x,\omega ) \partial _{\sigma }^{l_1}R_{1,N}(t,x,\sigma ' + \sigma ,\omega ) h_k^{(l_2)}(t + \sigma ' - \sigma + x\cdot \omega ) \textrm{d}t\textrm{d}x, \\&C^{l_1l_2} (\sigma ',\sigma ,\omega ) = \int _{\Omega }\int _{-T-\sigma '}^{T - \sigma '}q(t,x) \partial _{\sigma }^{l_1}R_{1,N}(t,x,\sigma ' + \sigma ,\omega ) \partial _{\sigma }^{l_2} R_{2,N}(t,x,\sigma '-\sigma ,\omega ) \textrm{d}t\textrm{d}x\\&E_{kj}^{l_1l_2} (\sigma ',\sigma ,\omega ) = \int _{\Omega }\int _{-T-\sigma '}^{T - \sigma '}Q_j^k(t,x,\omega ) h_j^{(l_1)}(t + \sigma ' + \sigma - x\cdot \omega ) h_k^{(l_2)}(t + \sigma ' - \sigma + x\cdot \omega ) \textrm{d}t\textrm{d}x. \end{aligned}$$

Then,

$$\begin{aligned} \tilde{M} q (\sigma ',\sigma ,\omega ) = \sum _{k,j=0}^N \left( E_{kj}^{00} (\sigma ',\sigma ,\omega ) + A_j^{00}(\sigma ',\sigma ,\omega ) + B_k^{00}(\sigma ',\sigma ,\omega ) + C^{00} (\sigma ',\sigma ,\omega ) \right) \end{aligned}$$

Next, we note that

$$\begin{aligned}  &   \partial _\sigma ^m E_{kj}^{00} \in \text {span} \left\{ \{E_{kj}^{l_1l_2}\}_{\begin{array}{c} l_1+l_2 = m \\ l_1<j, l_2<k \end{array}}, \; \{\partial _\sigma ^{m - k - l} A_{kj}^l\}_{\begin{array}{c} k + l\le m, l<j \end{array}}, \; \{\partial _\sigma ^{m - j - l} B_{kj}^l\}_{\begin{array}{c} j + l\le m, l<j \end{array}} \right\} ,\\  &   \partial _\sigma ^m A_j^{00}, \; \partial _\sigma ^m B_j^{00} \in \text {span} \left\{ \left\{ A_j^{l,m-l}, \; A_j^{l,m-l} \right\} _{l<j}, \; \left\{ \partial _\sigma ^{m-j-l}A_j^l, \; \partial _\sigma ^{m-j-l}B_j^l\right\} _{m\ge j + l} \right\} ,\\  &   \partial _\sigma ^m C^{00} \in \text {span} \left\{ \left\{ C^{l,m-l}\right\} _{l\le m} \right\} . \end{aligned}$$

Therefore,

27 $$\begin{aligned} \Vert \tilde{M}_{11}q(\sigma ',\cdot ,\cdot )\Vert _{L^2(\mathbb {S}^{n-1}_\omega ; H^{(n-1)/2}(\Sigma _\sigma ))} \le \sum M_{kj}^{\theta }, \end{aligned}$$

where the sum is taking over all *j*, $k\le N$ and the vectors $\theta = (\gamma _1,\gamma _2, \eta _1,\eta _2, \tau _1,\tau _2,\nu _1,\nu _2,\mu _1,\mu _2)$ such that

$$\begin{aligned}  &   \eta _1< k, \qquad \eta _2,\gamma _2, \tau _2 < j,\\  &   \gamma _1+\gamma _2 + j, \; \eta _1 + \eta _2, \; \tau _1+\tau _2, \; \nu _1+\nu _2+j, \; \mu _1 + \mu _2\le \frac{n-1}{2}, \end{aligned}$$

and

$$\begin{aligned} M_{kj}^{\theta }&= \Vert \partial _\sigma ^{\gamma _1}A_{kj}^{\gamma _2}(\sigma ',\cdot ,\cdot )\Vert _{L^2(\Sigma _\sigma \times \mathbb {S}_\omega ^{n-1})} + \Vert \partial _\sigma ^{\gamma _1}B_{jk}^{\gamma _2}(\sigma ',\cdot ,\cdot )\Vert _{L^2(\Sigma _\sigma \times \mathbb {S}_\omega ^{n-1})}\\&\quad + \Vert \partial _\sigma ^{\nu _1}A_{j}^{\nu _2}(\sigma ',\cdot ,\cdot )\Vert _{L^2(\Sigma _\sigma \times \mathbb {S}_\omega ^{n-1})} + \Vert \partial _\sigma ^{\nu _1}B_{j}^{\nu _2}(\sigma ',\cdot ,\cdot )\Vert _{L^2(\Sigma _\sigma \times \mathbb {S}_\omega ^{n-1})}\\&\quad + \Vert A_{j}^{\tau _2\tau _1}(\sigma ',\cdot ,\cdot )\Vert _{L^2(\Sigma _\sigma \times \mathbb {S}_\omega ^{n-1})} + \Vert B_{j}^{\tau _1\tau _2}(\sigma ',\cdot ,\cdot )\Vert _{L^2(\Sigma _\sigma \times \mathbb {S}_\omega ^{n-1})}\\&\quad + \Vert C^{\mu _1\mu _2}(\sigma ',\cdot ,\cdot )\Vert _{L^2(\Sigma _\sigma \times \mathbb {S}_\omega ^{n-1})} + \Vert E^{\eta _1\eta _2}(\sigma ',\cdot ,\cdot )\Vert _{L^2(\Sigma _\sigma \times \mathbb {S}_\omega ^{n-1})} . \end{aligned}$$

As we noted in the proof of Lemma 3.4, $\partial _s^{l}R_{1,N}$ satisfies

28 $$\begin{aligned} (\partial _t^2-\Delta +q_1)D_{s}^{l}R_{1,N}(t,x,s,\omega )= - [(\partial _t^2-\Delta +q_1 )a_{1,N}](t,x,\omega )h_{N}^{l}(s + t - x\cdot \omega ) \end{aligned}$$

and

29 $$\begin{aligned} D_{s}^{l}R_{1,N}(t,x,s,\omega ) \arrowvert _{t < -s - \rho } = 0. \end{aligned}$$

The similar property holds for $R_{2,N}$. Therefore, the same steps we used to prove Lemma 3.5, will give

30 $$\begin{aligned}  &   \Vert \partial _\sigma ^{\gamma _1}A_{kj}^{\gamma _2}(\sigma ',\cdot ,\cdot )\Vert _{L^2(\Sigma _\sigma \times \mathbb {S}_\omega ^{n-1})} + \Vert \partial _\sigma ^{\gamma _1}B_{jk}^{\gamma _2}(\sigma ',\cdot ,\cdot )\Vert _{L^2(\Sigma _\sigma \times \mathbb {S}_\omega ^{n-1})}\nonumber \\  &   \quad + \Vert \partial _\sigma ^{\nu _1}A_{j}^{\nu _2}(\sigma ',\cdot ,\cdot )\Vert _{L^2(\Sigma _\sigma \times \mathbb {S}_\omega ^{n-1})} + \Vert \partial _\sigma ^{\nu _1}B_{j}^{\nu _2}(\sigma ',\cdot ,\cdot )\Vert _{L^2(\Sigma _\sigma \times \mathbb {S}_\omega ^{n-1})}\nonumber \\    &   \quad \le \varepsilon C_{\Omega } \Vert q\Vert _{L^\infty (\mathbb {R}; L^2(\mathbb {R}^n))} \end{aligned}$$

for any $$\sigma '\in \mathbb {R}$$.

Next, we estimate

$$\begin{aligned}  &   |B_{j}^{\tau _1\tau _2}(\sigma ',\sigma ,\omega )| \le \Vert a_{2,j}\Vert _{L^{\infty }(\mathbb {R}\times \Omega \times \mathbb {S}^{n-1})} \\  &   \quad \times \int _{\Omega } \Vert q(\cdot , x)\Vert _{L^\infty (\mathbb {R})} \int _{-T}^T |\partial _{\sigma }^{\tau _1}R_{1,N}(t-\sigma ',x,\sigma ' + \sigma ,\omega )| h_j^{(\tau _2)}(t - \sigma + x\cdot \omega ) \textrm{d}t\textrm{d}x. \end{aligned}$$

Due to Lemma 3.4, it follows

$$\begin{aligned}  &   \Vert B_{j}^{\tau _1\tau _2}(\sigma ',\cdot ,\cdot )\Vert _{L^\infty (\Sigma \times \mathbb {S}^{n-1})}\\  &   \quad \le C_\Omega \Vert q\Vert _{L^\infty (\mathbb {R};L^2(\Omega ))} \sup _{\sigma \in \Sigma }\sup _{\omega \in \mathbb {S}^{n-1}} \sup _{x\in \Omega }\sup _{t\in [-T,T]} |\partial _{\sigma }^{\tau _1}R_{1,N}(t-\sigma ',x,\sigma ' + \sigma ,\omega )|. \end{aligned}$$

To estimate the right-hand side, we repeat some steps of Lemma 3.4. Since $\partial _s^{l}R_{1,N}$ satisfies (28) and (29), Lemma 3.3 gives

$$\begin{aligned}&\sup _{\sigma \in \Sigma } \sup _{t\in [-T,T]} |\partial _{\sigma }^{\tau _1}R_{1,N}(t-\sigma ',x,\sigma ' + \sigma ,\omega )|\\  &\quad \le C_\Omega \int _{-\sigma -\sigma ' - \rho }^{T-\sigma '} \Vert A_{1,N}(\tau ,\cdot ,\omega )h_N^{\tau _1}(T+\sigma - (\cdot )\cdot \omega \Vert _{H^K(\mathbb {R}^n)}\text {d}\tau , \end{aligned}$$

where $K = (n-1)/2 + 1$ and $A_{1,N}$ is the function defined by (18). Let

$$\begin{aligned} z=\sup _{\sigma \in \Sigma }T+\sigma . \end{aligned}$$

Due to (22),

$$\begin{aligned} \sup _{\sigma \in \Sigma } \sup _{t\in [-T,T]} |\partial _{\sigma }^{\tau _1}R_{1,N}(t-\sigma ',x,\sigma ' + \sigma ,\omega )| \le C_{\Omega } \sup _{\tau \in \mathbb {R}}\Vert A_{1,N}(\tau ,\cdot ,\omega )\Vert _{H^K(\tilde{\Omega })} \end{aligned}$$

for some compact $\tilde{\Omega }$ which depends only on $\Omega$. Then, (23) gives

$$\begin{aligned} \sup _{\sigma \in \Sigma } \sup _{t\in [-T,T]} |\partial _{\sigma }^{\tau _1}R_{1,N}(t-\sigma ',x,\sigma ' + \sigma ,\omega )| \le C_{\Omega } \Vert q_1\Vert _{C^{L + \frac{n-1}{2}+3+2N}(\mathbb {R}\times \bar{\Omega })} \le \varepsilon C_\Omega , \end{aligned}$$

and hence,

$$\begin{aligned} \Vert B_{j}^{\tau _1\tau _2}(\sigma ',\cdot ,\cdot )\Vert _{L^\infty (\Sigma \times \mathbb {S}^{n-1})} \le \varepsilon C_\Omega \Vert q\Vert _{L^\infty (\mathbb {R};L^2(\Omega ))}. \end{aligned}$$

Similarly, this estimate holds also for $A_{j}^{\tau _2\tau _1}$, $C^{\mu _1\mu _2}$, and $E^{\eta _1\eta _2}$. Hence, from (27) and (30), we obtain (26). $\square$

Finally, we prove Theorem 2.1.

#### Proof of Theorem 2.1

Let *C* be a constant form Lemma 3.1 and $C_\Omega$ be a common constant from Lemmas 3.5 and 3.6. Let us fix $0<\varepsilon <1$ such that $1/C - 3\varepsilon C_\Omega >0$. Next, we choose $k\in \mathbb {N}$ as large as Lemmas 3.5 and 3.6 require. Then, under conditions $\Vert q_1\Vert _{C^k(\mathbb {R}\times \bar{\Omega })}$, $\Vert q_2\Vert _{C^k(\mathbb {R}\times \bar{\Omega })}<\varepsilon$, Lemmas 3.1, 3.5, and 3.6 imply

$$\begin{aligned} \Vert \tilde{M}q\Vert _{L^\infty (\mathbb {R}_{\sigma '}; L^2(\mathbb {S}_\omega ^{n-1}; H^{(n-1)/2}(\Sigma _\sigma )))} \ge \frac{1}{C}\Vert q\Vert _{L^\infty (\mathbb {R}; L^2(\mathbb {R}^n))} - 3 \varepsilon C_{\Omega } \Vert q\Vert _{L^\infty (\mathbb {R}; L^2(\mathbb {R}^n))}. \end{aligned}$$

This completes the proof. $\square$

#### Remark 3.2

We want to emphasize on some subtle moment in the proof. The integration in (6) happens inside the wedge in Fig. 1, which, intersected with the cylinder $|x|\le \rho$, is compact. On the other hand, $M_{00}q$ integrates over the “line segment” (a hyperplane) there only while the other integrals integrate *q* inside the whole wedge. In order to absorb the $\tilde{M}_{10}q$, etc., terms, we need them to be small in *q*, which they are but they depend on *q* over a set larger than the one needed in $\tilde{M}_{00}q$. This arguments still works because we actually extend the estimates to *q* everywhere in the *t* variable by taking a supremum in $\sigma '$ above. On the other hand, if we wanted to establish local stability, like estimating *q* for *t* over a finite time interval having finite time backscattering data, that would have been be a problem.

## Appendix A. Scattering Theory for Time-Dependent Potentials

We recall the basics of the scattering theory for time-dependent perturbations of the wave equation by restricting it to time-dependent potentials. We follow Cooper and Strauss [3, 4], where it was introduced for moving obstacles, and its adaptation to time-dependent potentials in [20]. Some of the statements below are new, however, like Theorem A.4 and Theorem A.5. This theory is a natural extension of (a part of) the Lax-Phillips scattering theory. We consider the wave equation (3) with a smooth time-dependent potential *q*(*t*, *x*) supported in the cylinder $\mathbb {R}\times \overline{B(0,R)}$. We assume $n\ge 3$, odd to avoid working with the non-local translation representation when *n* is even.

### Lax-Phillips Formalism About the Wave Equation

The natural Cauchy problem for the wave equation is the following

31 $$\begin{aligned} (\partial _t^2-\Delta )u=0, \quad (u,u_t)|_{t=0} = (f_1,f_2). \end{aligned}$$

We convert the wave equation into a system by setting $\boldsymbol{u}(t)=(u,u_t)$; then

32 $$\begin{aligned} \partial _t \boldsymbol{u} = A\boldsymbol{u}, \quad A:= \begin{pmatrix} 0 & \quad \text {Id}\\ \Delta & \quad 0 \end{pmatrix}. \end{aligned}$$

We use boldface to denote vector-valued functions not necessarily of the type $(u,u_t)$ if there is no background scalar function *u*(*t*, *x*) present. In particular, $\boldsymbol{u}(t)$ in Definition A.1 below is not necessarily of that form.

The natural energy space of states of finite energy is defined as the completion of $C_0^\infty (\mathbb {R}^n) \times C_0^\infty (\mathbb {R}^n)$ under the energy norm

$$\begin{aligned} \Vert \boldsymbol{f}\Vert ^2_{\mathcal {H}}= \frac{1}{2}\int \left( |\nabla f_1|^2+|f_2|^2 \right) \textrm{d} x, \quad \boldsymbol{f}:=(f_1,f_2). \end{aligned}$$

In particular, the first term defines the Dirichlet space $H_D(\mathbb {R}^n)$ with norm $\Vert \nabla f\Vert _{L^2}$. When $n\ge 3$, they are locally in $L^2$, as it follows from the Poincaré inequality. The operator *A* naturally extends to a skew-self-adjoint one (i.e., $\textrm{i}A$ is self-adjoint) on $\mathcal {H}$. Then, by Stone’s theorem, $U_0(t) = e^{tA}$ is a well-defined strongly continuous unitary group, and the solution of (32) is given by $\boldsymbol{u}(t) = U_0(t)\boldsymbol{f}$. The unitarity means energy conservation, in particular.

We define the local energy space $\mathcal {H}_{\text {loc}}$ in the usual way:

$$\begin{aligned} \mathcal {H}_{\text {loc}} = \{\boldsymbol{f}: \; \phi \boldsymbol{f} \in \mathcal {H} \text { for each } \phi \in C_0^\infty (\mathbb {R}^n)\}. \end{aligned}$$

By the finite speed of propagation, the Cauchy problem (31) has a well-defined solution in $\mathcal {H}_{\text {loc}}$ if the Cauchy data $\boldsymbol{f}$ is in $\mathcal {H}_{\text {loc}}$ only. We view those solutions as ones with (possibly) infinite energy but locally finite one. Then, $\boldsymbol{u}\in C(\mathbb {R};\; \mathcal {H}_{\text {loc}})$ and the wave equation is solved in distribution sense. One can easily extend this to distributions.

### Existence of Dynamics

By [9], see also [17, X.12], the solution to

$$\begin{aligned} (\partial _t^2-\Delta +q(t,x))u=0,\quad (u,u_t)|_{t=s}=(f_1,f_2) \end{aligned}$$

is given by $\boldsymbol{u}(t)= U(t,s)\boldsymbol{f}$, where $\boldsymbol{f}=(f_1,f_2)$ and *U*(*t*, *s*) is a two-parameter strongly continuous group of bounded operators with the properties

(i) $U(t,s)U(s,r) = U(t,r)$ for all *t*, *s*, *r*; and $U(t,t)=\text {Id}$,
(ii) $$\Vert U(t,s)\Vert \le \exp \left\{ C|t-s|\sup _{s\le \tau \le t, \; x\in \mathbb {R}^n}|q(\tau ,x)| \right\} $$,
(iii) for any $\boldsymbol{f}\in D(A)$, we have $U(t,s)\boldsymbol{f}\in D(A)$ and
  33 $$\begin{aligned} \frac{\textrm{d}}{\textrm{d} t} U(t,s)\boldsymbol{f} = (A-Q(t))U(t,s)\boldsymbol{f}, \quad \frac{\textrm{d}}{\textrm{d} s} U(t,s)\boldsymbol{f} = -U(t,s)(A-Q(s))\boldsymbol{f}, \end{aligned}$$

where $Q(t)\boldsymbol{f}= (0,q(t,\cdot )f_1)$ (and *Q*(*t*) is clearly bounded).

The two-parameter semi-group admits the expansion

34 $$\begin{aligned} U(t,s) = U_0(t-s)+\sum _{k=1}^\infty V_k(t,s), \end{aligned}$$

where

$$\begin{aligned} V_k(t,s)\boldsymbol{f}&= (-1)^k \int _s^t \textrm{d} s_1 \int _s^{s_1} \textrm{d} s_k \dots \int _s^{s_{k-1}}\textrm{d} s_k\\&\quad \times U_0(t-s_1)Q(s_1) \dots U_0(s_{k-1}-s_k) Q(s_k)U_0(s_k-s)\boldsymbol{f}, \quad k\gg 1. \end{aligned}$$

This expansion is an iterated version of the Duhamel’s formula

35 $$\begin{aligned} U(t,s)&= U_0(t-s)+ \int _s^t U(t,\sigma )Q(\sigma )U_0(\sigma -s) \,\textrm{d} \sigma \nonumber \\&= U_0(t-s)+ \int _s^t U_0(t-\sigma )Q(\sigma )U(\sigma ,s)\,\textrm{d} \sigma . \end{aligned}$$

The convergence of (34) follows from the estimate

$$\begin{aligned} \Vert V_k(t,s)\Vert \le \frac{|t-s|^k}{k!} \left( \sup _{s\le \tau \le t}\Vert Q(\tau )\Vert \right) ^k. \end{aligned}$$

In particular, we get that we still have the finite speed of propagation property:

$$\begin{aligned} {{\,\textrm{supp}\,}}U(t,s)\boldsymbol{f}\subset {{\,\textrm{supp}\,}}\boldsymbol{f}+B(0,|t-s|). \end{aligned}$$

As before, the finite speed of propagation allows us to extend *U*(*t*, *s*) to the space $\mathcal {H}_{\text {loc}}$ by a partition of unity.

Finally, notice that when *q* is time independent, then *U*(*t*, *s*) depends on the difference $t-s$ only, i.e., $U(t,s)=U(t-s)$ where *U* is a group. It is not unitary, however (unless $q=0$), in the space $\mathcal {H}$. If we redefine the energy norm by

$$\begin{aligned} \Vert \boldsymbol{f}\Vert ^2_{\mathcal {H}^q}= \int \left( |\nabla f_1|^2+q|f_1|^2+ |f_2|^2 \right) \textrm{d} x, \end{aligned}$$

(we need to know that it is a norm, however, and $q\ge 0$ suffices for that), then *U*(*t*) is unitary in $\mathcal {H}^q$.

### Plane Waves, Translation Representation and Asymptotic Wave Profiles of Free Solutions

The plane waves

$$\begin{aligned} \delta (t-\omega \cdot x) \end{aligned}$$

solve the wave equation, obviously. They can be thought of as plane waves propagating in the direction $\omega$ with speed one. If we replace *t* by $t+s$ there, we can think of *s* as the delay time. The plane wave above is the Schwartz kernel of the Radon transform

$$\begin{aligned} Rf(s,\omega ) = \int \delta (s-\omega \cdot x)f(x)\,\textrm{d} x= \int _{x\cdot \omega =s}f(x)\,\textrm{d} S_x. \end{aligned}$$

For any density $g(\omega ,s)$ (which can be a distribution as well), the superposition

$$\begin{aligned} u(t,x) := \int _{\mathbb {R}\times S^{n-1}}\delta (t+s-\omega \cdot x)g(s,\omega )\,\textrm{d} s\,\textrm{d} \omega = \int _{S^{n-1}} g(\omega \cdot x-t,\omega )\,\textrm{d}\omega \end{aligned}$$

is still a solution of the wave equation. The expression above can be recognized as the transpose $R'$ of the Radon transform applied to $g_t(s,\omega ):= g(s-t,\omega )$. It turns out that all solutions of the free wave equation in the energy space have that form.

Indeed, in [13], Lax and Phillips defined the *free translation representation* $\mathcal {R}: \mathcal {H}\rightarrow L^2(\mathbb {R}\times S^{n-1})$ as follows

$$\begin{aligned} k(s,\omega ) = \mathcal {R}\boldsymbol{f}(s,\omega ) = c_n(-\partial _s^{(n+1)/2} Rf_1+\partial _s^{(n-1)/2} Rf_2), \end{aligned}$$

where *R* is the Radon transform and $c_n=2^{-1}(2\pi )^{(1-n)/2}$, $c_n^-= 2^{-1}(-2\pi )^{(1-n)/2}$. The inverse is given by

36 $$\begin{aligned} \mathcal {R}^{-1}k(x) = 2c_n^-\int _{S^{n-1}}\left( -\partial _s^{(n-3)/2} k(x\cdot \omega ,\omega ), \; \partial _s^{(n-1)/2}k(x\cdot \omega ,\omega )\right) \textrm{d}\omega . \end{aligned}$$

The map $\mathcal {R}$ is unitary, and $(\mathcal {R}U_0(t)\mathcal {R}^{-1} k)(s,\omega ) = k(s-t,\omega )$, which explains the name. We also set

37 $$\begin{aligned} u^\sharp (s,\omega ) = (-1)^{(n-1)/2} k(s,\omega ) \end{aligned}$$

and call $u^\sharp$ the *asymptotic wave profile* of the solution $\boldsymbol{u}(t)= U_0(t)\boldsymbol{f}$. This name is justified by the theorem below, and it is the analog of the far free pattern for solutions of the free wave equation.

#### Theorem A.1

(Lax-Phillips, [13]). Let $\boldsymbol{u}(t)= U_0(t)\boldsymbol{f}$, $\boldsymbol{f}\in \mathcal {H}$. Then,

$$\begin{aligned} \int \Big | u_t-|x|^{- (n-1)/2 }u^\sharp \Big (|x|-t,\frac{x}{|x|}\Big ) \Big |^2\, \textrm{d} x \rightarrow 0, \quad \text {as } |t|\rightarrow \infty . \end{aligned}$$

#### Remark A.1

In [13], the factor $(-1)^{(n-1)/2}$ is missing from (37), i.e., $u^\sharp = k$. Cooper and Strauss in [3] found out that this factor must be present in (37).

### Outgoing Solutions and Their Asymptotic Wave Profiles

We follow here [2, 3]. Given *u*(*t*, *x*) (and only then), recall the notation $\boldsymbol{u}(t) :=(u(t,\cdot ),u_t(t,\cdot ))$, see (32).

#### Definition A.1

The function $\boldsymbol{u}(t)\in C(\mathbb {R};\;\mathcal {H}_{\text {loc}})$ is called outgoing if $\lim _{t\rightarrow -\infty } (\boldsymbol{u}(t),U_0(t) \boldsymbol{g})=0$ for each $\boldsymbol{g}\in C_0^\infty (\mathbb {R}^n) \times C_0^\infty (\mathbb {R}^n)$.

In this definition, $\boldsymbol{u}(t)$ does not need to be a solution of the wave equation (anywhere). On the other hand, if *u*(*t*, *x*) solves the wave equation in $|x|>\rho$ for some $\rho >0$, then, see [2, 3], *u* is outgoing if and only if for any $T\in \mathbb {R}$, $U_0(t-T)\boldsymbol{u}(T)=0$ in the forward cone $|x|<t-T-\rho$.

One simple example of non-trivial outgoing solutions (for $|x|>\rho )$ is the following. Let $p\in L^1_{\text {loc}}(\mathbb {R};\;L^2(\mathbb {R}^n))$ satisfy $p=0$ for $t<t_0$, where $t_0$ is fixed. Solve

38 $$\begin{aligned} (\partial _t^2-\Delta )u=p(t,x) \quad \text {in } \mathbb {R}\times \mathbb {R}^n. \end{aligned}$$

with Cauchy data

$$\begin{aligned} (u,u_t)|_{t=t_0}=(0,0). \end{aligned}$$

By Duhamel’s formula,

$$\begin{aligned} \boldsymbol{u}(t)= \int _{t_0}^t U_0(t-s)\boldsymbol{p}(s)\,\textrm{d} s, \quad \boldsymbol{p}(s):= (0,p(s,\cdot )). \end{aligned}$$

The latter is well defined in $\mathcal {H}_{\text {loc}}$ by finite speed of propagation. The solution for $t<t_0$ is just zero. Then, $\boldsymbol{u}$ is outgoing in a trivial way. Moreover, this is the unique outgoing solution of (38). Indeed, take the difference *v* of any two. Then, $\boldsymbol{v}(t)=U_0(t)\boldsymbol{f}$, where $\boldsymbol{f}$ is the initial condition. Then, $0=\lim _{t\rightarrow -\infty } (\boldsymbol{v}(t),U_0(t) \boldsymbol{g})= (\boldsymbol{f},\boldsymbol{g})$, for any test function $\boldsymbol{g}$; therefore, $\boldsymbol{f}=0$ and then $\boldsymbol{v}=0$.

This can be generalized as follows.

#### Theorem A.2

([2, 3]). Let $p\in L^1_{\text {loc}}(\mathbb {R};\;L^2(\mathbb {R}^n))$ and assume that for each *t*,

39 $$\begin{aligned} \lim _{T\rightarrow -\infty }\int _{T}^t U_0(-s)\boldsymbol{p}(s)\, \textrm{d} s \quad \text {exists in } \mathcal {H}_{\text {loc}}, \quad \boldsymbol{p}(s) := (0,p(s,\cdot )). \end{aligned}$$

Then, there exists a unique outgoing solution $\boldsymbol{u}\in C(\mathbb {R};\;\mathcal {H}_{\text {loc}})$ of (38) given by

$$\begin{aligned} \boldsymbol{u}(t)= \int _{-\infty }^t U_0(t-s)\boldsymbol{p}(s)\,\textrm{d} s. \end{aligned}$$

#### Remark A.2

Clearly, $p\in L^1((-\infty ,a);\;L^2(\mathbb {R}^n))$ for any *a* would guarantee the regularity assumption on *p* and (39). Also, the assumptions on *p* in the next theorem are enough.

#### Proof

The absolute convergence of the integral in ${H}^0_{\text {loc}}$ follows from the assumptions. To show that *u* is outgoing, for $\boldsymbol{g} \in C_0^\infty (\mathbb {R}^n)\times C_0^\infty (\mathbb {R}^n)$, consider

$$\begin{aligned} (\boldsymbol{u}(t), U_0(t)\boldsymbol{g})= \int _{-\infty }^t\left( U_0(t-s)\boldsymbol{p}(s), U_0(t)\boldsymbol{g} \right) \textrm{d} s = \int _{-\infty }^t\left( U_0(-s)\boldsymbol{p}(s), \boldsymbol{g} \right) \textrm{d} s. \end{aligned}$$

The latter converges to 0, as $t\rightarrow -\infty$ by assumption. $\square$

#### Theorem A.3

([2, 3]). Let $n\ge 3$ be odd. Let $p\in L^1_{\text {loc}}(\mathbb {R};\;L^2(\mathbb {R}^n))$ with $p(t,x)=0$ for $|x|>\rho$. Let *u* be the unique outgoing solution of (38).

- Then, there is a unique function $u^\sharp \in L_{\text {loc}}^2(\mathbb {R}\times S^{n-1})$ such that for all $R_1<R_2$ we have
  $$\begin{aligned} \int _{R_1+t<|x|<R_2+t} \left| u_t(t,x) - |x|^{-(n-1)/2} u^\sharp \left( |x|-t,\frac{x}{|x|}\right) \right| ^2\textrm{d} x\rightarrow 0, \quad \text {as } t\rightarrow \infty . \end{aligned}$$
- If $$p\in C_0^\infty (\mathbb {R}^{n+1})$$,
  40 $$\begin{aligned} u^\sharp (s,\omega ) = c_n^- \partial _s^{(n-1)/2}\int p(\omega \cdot x-s,x)\,\textrm{d} x. \end{aligned}$$
- The map $p\rightarrow u^\sharp$ is continuous.

#### Remark A.3

For general *p* as in the theorem, $u^\sharp$ is still given by (40) but the derivative is in distribution sense; by (b), the result is in $L_{\text {loc}}^2(\mathbb {R}\times S^{n-1})$. Another way to write (40) is

$$\begin{aligned} \int u^\sharp (s,\omega )\phi (s)\,\textrm{d} s = c_n \iint p(t,x) (\partial _s^{(n-1)/2} \phi ) (\omega \cdot x-s,x)\,\textrm{d} t\, \textrm{d} s, \quad \forall \psi (s)\in C_0^\infty (\mathbb {R}). \end{aligned}$$

#### Proof

Motivated by Theorem A.2, for fixed $R_1<R_2$, set

$$\begin{aligned} \boldsymbol{f} = \int _{-R_2-\rho }^{-R_1+\rho }U_0(-\tau )\boldsymbol{p}(\tau )\,\textrm{d} \tau , \quad \boldsymbol{v}(t) = U_0(t)\boldsymbol{f}. \end{aligned}$$

By Huygens’ principle, $\boldsymbol{v}(t)=\boldsymbol{u}(t)$ for $R_1+t< |x|<R_2+t$. Therefore, $\boldsymbol{v}$ does have an asymptotic wave profile, and $v^\sharp (s,\omega ) = u^\sharp (s,\omega )$ for $R_1<s<R_2$. On the other hand, we have a formula for $v^\sharp$, (36) and (37) which says

$$\begin{aligned} v^\sharp (s,\omega )&= (-1)^{(n-1)/2}(\mathcal {R}\boldsymbol{f})(s,\omega ) \\&= c_n^-\partial _s^{(n-1)/2} \int _{-R_2-\rho }^{-R_1+\rho } \int _{x\cdot \omega =s+\tau } p(x\cdot \omega -s,x)\, \textrm{d} S_x\, \textrm{d}\tau . \end{aligned}$$

Then,

$$\begin{aligned} u^\sharp (s,\omega )|_{R_1<s<R_2} = v^\sharp (s,\omega )= c_n^- \partial _s^{(n-1)/2}\int p(x\cdot \omega -s,x)\, \textrm{d} x. \end{aligned}$$

Since $R_1<R_2$ are arbitrary, this, combined with Theorem A.2, proves (a); and (b) for $p\in C_0^\infty$.

The proof of (c) is straightforward: use (40) and take Fourier transform w.r.t. *s*. In particular, we get that the map $p\rightarrow u^\sharp$ can be extended continuously in those spaces. $\square$

#### Remark A.4

We call $u^\sharp$ in the theorem the asymptotic wave profile of the unique outgoing solution of (38). Note that there are two cases where we defined such profiles: for free solutions in the energy space, see Theorem A.2, and in Theorem A.3 above, where *u* is in the energy space locally only, and solves (38) instead.

### Scattering Solutions

The scattering solutions $u^-$ and $u^+$ were introduced in Sect. 3 as the solutions of (3), and (5), respectively. Since they involve distributions, not necessarily in the energy spaces (even locally), we proceed as follows. We can think of $(u(t,x;s,\omega ), u_t(t,x;s,\omega ))$ as distribution in the $(s,\omega )$ variables with values in $\mathcal {H}_{\text {loc}}$. It is more convenient, however, to do the following. Let $h_j(t)=h(t)t^j/j!$, $j=1,2\dots$, where *h* is the Heaviside function; and we also set $h_{-1}=\delta$ . Then, $h_j'=h_{j-1}$, $j=0,1,2,\dots$. To define $u^-$ eventually, we solve

41 $$\begin{aligned} (\partial _t^2-\Delta +q(t,x))\Gamma =0, \quad \Gamma |_{t<-s-\rho }= h_1(t+s-x\cdot \omega ) \end{aligned}$$

first (notice that $h_1(t+s-x\cdot \omega )$ is locally in the energy space now), set

$$\begin{aligned} \Gamma _{\text {sc}} = \Gamma - h_1(t+s-x\cdot \omega ), \end{aligned}$$

compute the asymptotic wave profile $\Gamma ^\sharp (s',\omega ';s,\omega )$ of $\Gamma _{\text {sc}}$, and differentiate the result twice w.r.t. *s* to get the analog of the scattering amplitude. In particular, then

42 $$\begin{aligned} u(t,x;s,\omega ) = \partial _s^2\Gamma (t,x;s,\omega ), \quad u_{\text {sc}}^-(t,x;s,\omega ) = \partial _s^2\Gamma _{\text {sc}}(t,x;s,\omega ). \end{aligned}$$

will be well defined as distributions.

In a similar way, one can construct the scattering solutions $u^+$ which look like plane waves as $t\rightarrow +\infty$, instead of $t\rightarrow -\infty$. They would solve (5). Performing the change of variables $\tilde{t}=-t$ (time reversal), $\tilde{s}=-s$, $\tilde{\omega }=-\omega$, we see that $u^+(t,x;s,\omega ) =\tilde{u}^+(-t,x;-s,-\omega )$, where $\tilde{u}$ is related to $\tilde{q}(t,x):=q(-t,x)$. The regularized version, $\Gamma ^+$, can be constructed as in (41) with the condition $\Gamma ^+ = h_1(-t-s+x\cdot \omega )$ for $t>-s+\rho$. The right-hand side of this condition then would be supported outside $\mathbb {R}\times B(0,R)$ for $t>-s+\rho$. Then, we define $u^+$ and $u^+_{\text {sc}}$ as in (42).

By the finite speed of propagation property

43 $$\begin{aligned} {{\,\textrm{supp}\,}}u^-(\cdot ,\cdot ;s,\omega ) \subset \{t+s-x\cdot \omega \ge 0 \}, \quad {{\,\textrm{supp}\,}}u^+(\cdot ,\cdot ;s,\omega ) \subset \{t+s-x\cdot \omega \le 0 \}. \end{aligned}$$

Next theorem generalizes Theorem A.3.

#### Theorem A.4

Let *p* be as in Theorem A.3. Set

$$\begin{aligned} \boldsymbol{u}(t): = \int _{-\infty }^t U(t,s) \boldsymbol{p}(s)\,\textrm{d} s. \end{aligned}$$

Then, $\boldsymbol{u}\in C(\mathbb {R};\;\mathcal {H}_{\text {loc}})$ is outgoing and has an asymptotic wave profile $u^\sharp (s,\omega )$ given by

$$\begin{aligned} u^\sharp (s,\omega ) = c_n^- \partial _s^{(n-1)/2}\int p(t,x) u^+ (t,x;s,\omega )\,\textrm{d} t\,\textrm{d} x. \end{aligned}$$

#### Proof

By (35),

44 $$\begin{aligned} \boldsymbol{u}(t)&= \int _{-\infty }^t U_0(t-s) \boldsymbol{p}(s)\,\textrm{d} s + \int _{-\infty }^t\int _s^t U_0(t-\sigma )Q(\sigma ) U(\sigma ,s) \boldsymbol{p}(s)\,\textrm{d} \sigma \,\textrm{d} s\nonumber \\&= \int _{-\infty }^t U_0(t-s) \boldsymbol{p}(s)\,\textrm{d} s + \int _{-\infty }^t\int _{-\infty }^\sigma U_0(t-\sigma )Q(\sigma ) U(\sigma ,s) \boldsymbol{p}(s)\,\textrm{d} s\,\textrm{d} \sigma . \end{aligned}$$

Then, we are in the situation of Theorem A.3 with $\boldsymbol{p}(t)$ there replaced by

45 $$\begin{aligned} \tilde{\boldsymbol{p}}(t):= \boldsymbol{p}(t)+ \boldsymbol{p}_1(t), \quad \boldsymbol{p}_1(t):= Q(t)\int _{-\infty }^t U(t,s) \boldsymbol{p}(s)\,\textrm{d} s= Q(t)\boldsymbol{u}(t). \end{aligned}$$

Then, $\boldsymbol{u}$ has an asymptotic wave profile $u^\sharp (s',\omega ')$ satisfying

46 $$\begin{aligned} u^\sharp (s',\omega ') = c_n^- \partial _s^{(n-1)/2}\int \tilde{p}(t,x)\delta (t+s'-\omega '\cdot x)\,\textrm{d} x\,\textrm{d} t. \end{aligned}$$

The first term on the right-hand side of (44) is handled by Theorem A.3. We analyze the second term below, which we call $\boldsymbol{u}_1(t)$. By Theorem A.3 again, its asymptotic wave profile is given by

47 $$\begin{aligned} u_1^\sharp (s',\omega ')&= c_n^- \partial _s^{(n-1)/2}\int \Big [ Q(t,x)\int _{-\infty }^t U(t,x;s,y) \boldsymbol{p}(s,y)\,\textrm{d} s\,\textrm{d} y\Big ]_2\, \nonumber \\  &\quad {\delta }(t+s'-\omega '\cdot x)\,\textrm{d} t\,\textrm{d} x\nonumber \\&= c_n^- \partial _s^{(n-1)/2} \int K(s',\omega ';s,y) p(s,y)\,\textrm{d} s\,\textrm{d} y, \end{aligned}$$

where the last identity defines *K*, i.e.,

48 $$\begin{aligned} K(s',\omega ';s,y)= \int ^{\infty }_s \int q(t,x) U_{12}(t,x;s,y) \delta (t+s'-\omega '\cdot x)\,\textrm{d} x\,\textrm{d} t. \end{aligned}$$

By (33),

$$\begin{aligned} (-\partial _s + A_y'- Q'(s)) U'(t,x;s,y)=0, \end{aligned}$$

where *A* is the operator defined by (32) and the primes denote transpose operators in distribution (not in energy space) sense. This equality can be written also as

$$\begin{aligned} \begin{pmatrix} -\partial _s & \quad \Delta _y-q(s)\\ \text {Id}& \quad -\partial _s \end{pmatrix}U'(t,x;s,y) =0. \end{aligned}$$

In particular,

49 $$\begin{aligned} (\partial _s^2-\Delta _y+q(s)) U_{12}(t,x;s,y)=0. \end{aligned}$$

Differentiate *K* in (48) to obtain

$$\begin{aligned} \partial _s K(s',\omega ';s,y)= \int ^{\infty }_s \int q(t,x) \partial _s U_{12}(t,x;s,y) \delta (t+s'-\omega '\cdot x)\,\textrm{d} x\,\textrm{d} t \end{aligned}$$

because $U_{12}(s,x;s,y)=0$. Differentiate again:

$$\begin{aligned} \partial _s^2 K(s',\omega ';s,y)&= \int ^{\infty }_s \int q(t,x) \partial _s^2 U_{12}(t,x;s,y) \delta (t+s'-\omega '\cdot x)\,\textrm{d} x\,\textrm{d} t \\&\quad -q(s,x)\delta (s+s'-\omega '\cdot y). \end{aligned}$$

Then, by (49) and (48),

50 $$\begin{aligned} (\partial _s^2-\Delta _y+q(s)) K(s',\omega ';s,y)= -q(s,x)\delta (s+s'-\omega '\cdot y). \end{aligned}$$

On the support of the integrand in (48), we have $t+s'<\rho$, $s<t$. Therefore,

51 $$\begin{aligned} K(s',\omega ';s,y)|_{s>-s'+\rho }=0. \end{aligned}$$

Therefore, *K* solves (50), (51), which is the same problem solved by $u^+_{\text {sc}}(s',\omega ';s,y)$, see (5). Therefore, $K=u^+_{\text {sc}}$.

Going back to (46) and (45), we see that

$$\begin{aligned} u^\sharp (s',\omega ')&= c_n^- \partial _s^{(n-1)/2}\int \left( p(t,x)+p_1(t,x) \right) \delta (t+s'-\omega '\cdot x)\,\textrm{d} x\,\textrm{d} t\\&= c_n^- \partial _s^{(n-1)/2}\int p(t,x) ) \left( \delta (t+s'-\omega '\cdot x)+ u^+_{\text {sc}}(s',\omega ';t,x)\right) \,\textrm{d} x\,\textrm{d} t\\&= c_n^- \partial _s^{(n-1)/2}\int p(t,x) ) u^+(s',\omega ';t,x) \,\textrm{d} x\,\textrm{d} t, \end{aligned}$$

where we used (47) and the identity $K=u^+_{\text {sc}}$ we just derived. $\square$

### The Scattering Amplitude and the Scattering Kernel

Let $\Gamma$ solve (41). Since the Cauchy data $(h_1(t+s-x\cdot \omega ), h_0(t+s-x\cdot \omega ))$, for say, $t=-s-\rho -1$, is in $\mathcal {H}_{\text {loc}}$, a solution $(\Gamma ,\Gamma _t)$ with locally finite energy exists. Then, $\Gamma _{\text {sc}}$ is clearly outgoing. It solves the Cauchy problem

$$\begin{aligned} (\partial _t^2-\Delta )\Gamma _{\text {sc}}= -q\Gamma ,\quad \Gamma _{\text {sc}}|_{t<-s-\rho }= 0. \end{aligned}$$

By Theorem A.3, $\Gamma _{\text {sc}}$ has an asymptotic wave profile $\Gamma _{\text {sc}}^\sharp$ given by

$$\begin{aligned} \Gamma _{\text {sc}}^\sharp (s',\omega ';s,\omega )&= -c_n^-\partial _{s'}^{(n-1)/2} \int q (x\cdot \omega '-s' ,x )\Gamma (x\cdot \omega '-s' ,x;s,\omega )\, \textrm{d} x\\&= -c_n^-\partial _{s'}^{(n-1)/2} \int q (t ,x )\Gamma (t ,x;s,\omega )\delta (t+s'- x\cdot \omega ' )\, \textrm{d} t\,\textrm{d} x. \end{aligned}$$

Differentiate twice w.r.t. *s*, see (42), to get

$$\begin{aligned} u^{-,\sharp }_{\text {sc}} (s',\omega ';s,\omega )= -c_n^-\partial _{s'}^{(n-1)/2} \int q (t,x )u^-(t,x;s,\omega )\delta (t+s'- x\cdot \omega ' )\, \textrm{d} t\,\textrm{d} x. \end{aligned}$$

#### Definition A.2

The scattering amplitude $A^\sharp$ is given by

$$\begin{aligned} A^\sharp (s',\omega ';s,\omega ) = \int q (t ,x )u^-(t ,x;s,\omega )\delta (t+s'- x\cdot \omega ' )\, \textrm{d} t\,\textrm{d} x, \end{aligned}$$

where $$u^-$$ solves (3).

By the finite speed of propagation, $u^-(t,x;s,\omega ) = 0$ for $x\cdot \omega >t+s$. Therefore, the integrand vanishes outside of the region $x\cdot (\omega -\omega ')\le s-s'$. The l.h.s. has a lower bound $-2\rho$ on ${{\,\textrm{supp}\,}}q$; therefore,

$$\begin{aligned} {{\,\textrm{supp}\,}}A^\sharp \subset \{ s'\le s+\rho |\omega -\omega '|\}\subset \{ s'\le s+2\rho \}. \end{aligned}$$

Note that $A^\sharp$ and $u^\sharp _{\text {sc}} = -c_n^-\partial _{s'}^{(n-1)/2} A^\sharp$ can be reconstructed from each other thanks to that support property.

Since the perturbed dynamics is a two-parameter group, we need to generalize the notion of the wave operators and the scattering operator.

#### Definition A.3

The wave operators $\Omega _-$ and $W_+$ in $\mathcal {H}$ are defined as the strong limits

$$\begin{aligned} \Omega _- = \text {s\,-}\lim _{t\rightarrow -\infty }U(0,t)U_0(t), \quad W_+ \boldsymbol{f}= \lim _{t\rightarrow \infty }U_0(-t)U(t,0)\boldsymbol{f}; \quad \boldsymbol{f}\in \text {Ran}\;\Omega _-, \end{aligned}$$

if they exist and define continuous operators. In the latter case, the scattering operator *S* is defined by

$$\begin{aligned} S= W_+\Omega _-. \end{aligned}$$

This definition also makes sense for $\boldsymbol{f} \in \mathcal {H}_{\text {comp}}$, where $\mathcal {H}_{\text {comp}}$ denotes the subspace of $\mathcal {H}$ consisting of compactly supported functions, with $S\boldsymbol{f}$ taking values possibly in $\mathcal {H}_{\text {loc}}$.

#### Theorem A.5

- The wave operator $\Omega _-: \mathcal {H}_{\text {comp}} \rightarrow \mathcal {H}$ exists and
  52 $$\begin{aligned} U(t,0)\Omega _-\boldsymbol{f} = 2c_n^-\int _{\mathbb {R}\times S^{n-1}} \boldsymbol{u}^-(t,x;s,\omega ) \partial _s^{(n-3)/2} (\mathcal {R}\boldsymbol{f})(s,\omega )\,\textrm{d} s\,\textrm{d}\omega . \end{aligned}$$
- The wave operator $W_+: \mathcal {H}\rightarrow \mathcal {H}_{\text {loc}}$ exists.
- The scattering operator $S:\mathcal {H}_{\text {comp}}\rightarrow \mathcal {H}_{\text {loc}}$ exists.

#### Proof

Choose $\boldsymbol{f}\in \mathcal {H}_{\text {comp}}$, so that $\boldsymbol{f}(x)=0$ for $|x|>R$ with some $R>0$. Let $k=\mathcal {R}\boldsymbol{f}$. Then, $k(s,\omega )=0$ for $|s|>R$. For $t<-R-\rho :=t_0$, $U(0,t)U_0(t)\boldsymbol{f}= U(0,t_0)U_0(t_0)\boldsymbol{f}$. In particular, the limit defining $\Omega _-\boldsymbol{f}$ exists trivially and $U(t,0)\Omega _-\boldsymbol{f} = U(t,t_0)U_0(t_0)\boldsymbol{f}$. The r.h.s. of the latter solves the perturbed wave equation and equals $U_0(t_0)\boldsymbol{f}= \mathcal {R}^{-1} k(\cdot -t_0,\cdot )$ for $t=t_0$. To prove (52), we need to show that the r.h.s. of (52), call it $\boldsymbol{v}(t)$, has the same initial condition for $t\le t_0$.

For $t\le t_0$, $u(t,x;s,\omega )= \delta (t+s-x\cdot \omega )$. Then, by (36),

$$\begin{aligned} v(t) = 2c_n^-\int _{\mathbb {R}\times S^{n-1}} \delta (t+s-x\cdot \omega )\partial _s^{(n-3)/2}k(s,\omega )\,\textrm{d} s\,\textrm{d}\omega = (\mathcal {R}^{-1}k)_1(\cdot -t,\cdot ), \end{aligned}$$

which proves (a).

To prove the existence of $W_+$ in (b), fix first $R>0$ and let $\boldsymbol{1}_{B(0,R)}$ be the characteristic function of that ball. By (35),

$$\begin{aligned} \boldsymbol{1}_{B(0,R)}U_0(-t) U(t,s) = \boldsymbol{1}_{B(0,R)}U_0(-s)+ \boldsymbol{1}_{B(0,R)} \int _s^t U_0(-\sigma )Q(\sigma )U(\sigma ,s)\,\textrm{d} \sigma . \end{aligned}$$

By Huygens’ principle, $\boldsymbol{1}_{B(0,R)} U_0(-\sigma )Q(\sigma )=0$ for $\sigma >R+\rho$. For $t>R+\rho$, then the integral above is independent of *t* and therefore the strong limit $\boldsymbol{1}_{B(0,R)}W_+$ exists in a trivial way, defining a unique element in $\mathcal {H}_{\text {loc}}$.

Part (c) follows from (a) and (b). $\square$

The scattering operator *S* on $\mathcal {H}$ exists (as a bounded operator) under some conditions, see the references in [20, sec. 3]. Then, $-c_n^-\partial _{s'}^{(n-1)/2} A^\sharp (s',\omega '; s,\omega )$ is the Schwartz kernel of $\mathcal {R}(S-\text {Id})\mathcal {R}^{-1}$. In the general case, we can consider the latter as the Schwartz kernel of the operator mapping asymptotic wave profiles instead of translation representations, see (37), as shown [20, Proposition 3.1].

#### Proposition 1

([2, 3, 20]). Let $\boldsymbol{f}\in C_0^\infty (\mathbb {R}^n) \times C_0^\infty (\mathbb {R}^n)$. Let $\boldsymbol{v}_0(t)= U_0(t)\boldsymbol{f}$, and let $\boldsymbol{v}(t)$ be the solution of (1) which equals $\boldsymbol{v}_0(t)$ for $t\ll 0$. Then, we have

$$\begin{aligned} v^\sharp (s',\omega ') = v_0^\sharp (s',\omega ')-c_n^-\partial _{s'}^{(n-1)/2}\int _{\mathbb {R}\times S^{n-1}} A^\sharp (s',\omega '; s,\omega ) v_0^\sharp (s,\omega )\,\textrm{d} s\,\textrm{d}\omega . \end{aligned}$$

The proof of the proposition is done by taking the asymptotic wave profile of (52) and applying Duhamel’s formula (35) first.

Finally, we will prove Proposition 3.1

#### Proof of Proposition 3.1

Start with

$$\begin{aligned} U_1(t,s)-U_2(t,s) = \int _s^t U_1(t,\sigma )(Q_1(\sigma )-Q_2(\sigma ))U_2(\sigma ,s)\,\textrm{d} \sigma , \end{aligned}$$

which can be obtained by applying the Fundamental Theorem of Calculus to $F(\sigma )= U_1(t,\sigma ) U_2(\sigma ,s)$ in the interval $\sigma \in [s,t]$. Apply $U_0(-s)\boldsymbol{f}$ on the right-hand, and take the (strong) limit $s\rightarrow -\infty$ to get

53 $$\begin{aligned}&U_1(t,0)\Omega _{1,-}\boldsymbol{f} -U_2(t,0)\Omega _{2,-}\boldsymbol{f}\nonumber \\  &\quad = \int _{-\infty }^t U_1(t,\sigma )(Q_1(\sigma )-Q_2(\sigma )) \left[ U_2(\sigma ,0) \Omega _{2,-}\boldsymbol{f}\right] \,\textrm{d} \sigma . \end{aligned}$$

For the left-hand side, and for the expression in the square brackets we will apply Theorem A.5(a). We take the asymptotic wave profile of (53) next applying Theorem A.4. Then, (6) is just that expression, written as a composition of Schwartz kernels, eventually applied to $\mathcal {R} \boldsymbol{f}$. $\square$

## Appendix B. A Weighted Radon Transform

We recall the definition of the weighted Euclidean Radon transform

$$\begin{aligned} R_\mu f (p,\omega ) = \int _{x\cdot \omega =p} \mu (x,\omega ) f(x) \textrm{d}x = \int _{\omega ^\perp } \mu (p\omega + y, \omega ) f(p\omega + y) \textrm{d}y. \end{aligned}$$

where $\mu \in C^\infty (\mathbb {R}\times \mathbb {S}^{n-1})$ is a weight. We will use the next result:

### Theorem B.1

Let $\Omega$ be an open, bounded set and $\mu \in C^\infty (\mathbb {R}\times \mathbb {S}^{n-1})$. Then, there is a constant $C_\Omega >0$, which depends only on $\Omega$, such that

$$\begin{aligned} \Vert R_\mu f\Vert _{L^2(\mathbb {S}_\omega ^{n-1}; H^{(n-1)/2}(\mathbb {R}_\sigma ))} \le C_\Omega \sup _{\omega \in \mathbb {S}^{n-1}} \Vert \mu (\cdot ,\omega )\Vert _{C^{2n - 2}(\bar{\Omega })} \Vert f\Vert _{L^2(\Omega )} \end{aligned}$$

for all $$f\in C_0^\infty (\Omega )$$.

To prove this, we need the following auxiliary lemmas.

### Lemma B.1

Let $\Omega \subset \mathbb {R}^n$ be an open, bounded set and $\nu$ be a function on $\mathbb {R}^n\times \mathbb {R}^n$ such that for any fixed $\xi \in \mathbb {R}^n$, $\nu (\cdot ,\xi ) \subset C^{n}(\bar{\Omega })$ with $\mathrm {supp\,}\nu (\cdot ,\xi ) \subset \Omega$. Then, the operator *V*, given by

$$\begin{aligned} V: f \rightarrow \int _{\mathbb {R}^n} e^{-ix\xi } \nu (x,\xi ) f(x)\textrm{d}x, \end{aligned}$$

satisfies

$$\begin{aligned} \Vert V\Vert _{L^2(\Omega ) \rightarrow L^2(\mathbb {R}^n)} \le C_\Omega \sup _{\xi \in \mathbb {R}^n} \Vert \nu (\cdot ,\xi )\Vert _{C^{n}(\bar{\Omega })}. \end{aligned}$$

### Proof

Throughout this proof, $C_\Omega$ will serve as a universal positive constant depending only on $\Omega$, which may vary from line to line. Let $f\in C_0^\infty (\Omega )$, then

$$\begin{aligned} |Vf(\xi )| = \left| \int _{\mathbb {R}^n} D_{x_n}\cdots D_{x_1} \left( \int _{-\infty }^{x_1}\cdots \int _{-\infty }^{x_n}f(y)e^{-iy\xi }\textrm{d}y_{n} \cdots \textrm{d}y_{1} \right) \nu (x,\xi ) \textrm{d}x\right| . \end{aligned}$$

By integration by parts, we obtain

$$\begin{aligned} |Vf(\xi )|\le &   \int _{\mathbb {R}^n} \left| \int _{-\infty }^{x_1}\cdots \int _{-\infty }^{x_n}f(y)e^{-iy\xi }\textrm{d}y_{n} \cdots \textrm{d}y_{1} \right| |D_{x_n}\cdots D_{x_1} \nu (x,\xi )| \textrm{d}x\\\le &   C_\Omega \sup _{\xi \in \mathbb {R}^n} \Vert \nu (\cdot ,\xi )\Vert _{C^{n}(\bar{\Omega })} \sup _{x\in \mathbb {R}^n} \left| \int _{\mathbb {R}^n} e^{-iy\xi }f(y) \chi _{\{z: z_k\le x_k\}}(y) \textrm{d}y\right| , \end{aligned}$$

where $\chi _A$ is the indicator function for a set *A*. Therefore, we estimate

$$\begin{aligned} \Vert Vf\Vert _{L^2(\mathbb {R}^n)}&\le C_\Omega \sup _{\xi \in \mathbb {R}^n} \Vert \nu (\cdot ,\xi )\Vert _{C^{n}(\bar{\Omega })} \sup _{x\in \mathbb {R}^n} \Vert \mathcal {F}[f\chi _{\{z: z_k\le x_k\}}]\Vert _{L^2(\Omega )}\\&\le C_\Omega \sup _{\xi \in \mathbb {R}^n} \Vert \nu (\cdot ,\xi )\Vert _{C^{n}(\bar{\Omega })} \sup _{x\in \mathbb {R}^n} \Vert f\chi _{\{z: z_k\le x_k\}}\Vert _{L^2(\Omega )}\\&\le C_\Omega \sup _{\xi \in \mathbb {R}^n} \Vert \nu (\cdot ,\xi )\Vert _{C^{n}(\bar{\Omega })} \Vert f\Vert _{L^2(\Omega )}. \end{aligned}$$

This completes the proof. $\square$

### Lemma B.2

Let $\omega \subset \mathbb {R}^n$ be an open bounded set and $\mu$, $\nu \in C^\infty (\mathbb {R}\times \mathbb {S}^{n-1})$. Then, there exists a constant $C_\Omega >0$, which depend only on $\Omega$, such that

$$\begin{aligned} |(R_\mu ^* R_\nu D^\gamma f, f)_{L^2(\mathbb {R}^n)}|\le &   C_\Omega \sup _{\omega \in \mathbb {S}^{n-1} }\Vert \mu (\cdot , \omega )\Vert _{C^{n+1}(\bar{\Omega })} \\    &   \sup _{\omega \in \mathbb {S}^{n-1}} \left\| \nu (\cdot , \omega ) \right\| _{C^{n -1 + |\gamma |}(\bar{\Omega })}\Vert f\Vert _{L^2(\Omega )}. \end{aligned}$$

for any $f\in C_0^\infty (\Omega )$ and multi-index any $\gamma$ with $0\le |\gamma |\le n-1$.

### Proof

Throughout this proof, $C_\Omega$ will serve as a universal positive constant depending only on $\Omega$, which may vary from line to line. Let $\chi \in C_0^\infty (\mathbb {R}^n)$ such that $\chi (x)=1$ for $x\in \Omega$. Then, for $f\in C_0^\infty (\Omega )$,

$$\begin{aligned} |(R_\mu ^* R_\nu D^\gamma f, f)_{L^2(\mathbb {R}^n)}|= &   |(\chi R_\mu ^* R_\nu D^\gamma (\chi f), f)_{L^2(\mathbb {R}^n)}|\\  \le &   \Vert \chi R_\mu ^* R_\nu D^\gamma (\chi f)\Vert _{L^2(\mathbb {R}^n)} \Vert f\Vert _{L^2(\mathbb {R}^n)}. \end{aligned}$$

Let us investigate the first multiplier on the right-hand side. By Proposition 5.8.3 in [23], $R^*_{\mu }R_{\nu }$ is a $\Psi \textrm{DO}$ of order $1-n$ with the amplitude given by

$$\begin{aligned} (2\pi - 1) \frac{\mu (x, \xi /|\xi |)\nu (y, \xi /|\xi |) + \mu (x, -\xi /|\xi |)\nu (y, -\xi /|\xi |)}{|\xi |^{n-1}}. \end{aligned}$$

Then,

$$\begin{aligned} \chi R_\mu ^* R_\nu D^\gamma (\chi f) = Af + Bf \end{aligned}$$

with

$$\begin{aligned} Af(x) = C_\Omega \int _{\mathbb {R}^n} \int _{\mathbb {R}^n} e^{i(x - y)\xi } \chi (x) \frac{\mu (x, \xi /|\xi |)\nu (y, \xi /|\xi |)}{|\xi |^{n-1}} D_y^\gamma (\chi (y) f(y)) \textrm{d}y \textrm{d}\xi \end{aligned}$$

and

$$\begin{aligned} Bf(x) = C_\Omega \int _{\mathbb {R}^n} \int _{\mathbb {R}^n} e^{i(x - y)\xi } \chi (x) \frac{\mu (x, -\xi /|\xi |)\nu (y, -\xi /|\xi |)}{|\xi |^{n-1}} D_y^\gamma (\chi (y) f(y)) \textrm{d}y \textrm{d}\xi . \end{aligned}$$

By integration by parts, we obtain

$$\begin{aligned} |Af(x)| \le C_\Omega \sum _{\alpha + \beta = \gamma } \left| \int _{\mathbb {R}^n} \int _{\mathbb {R}^n} e^{i(x - y)\xi } \chi (x) \xi ^\alpha \frac{\mu (x, \xi /|\xi |) D_y^\beta \nu (y, \xi /|\xi |)}{|\xi |^{n-1}} \chi (y) f(y) \textrm{d}y \textrm{d}\xi \right| . \end{aligned}$$

Let $\phi \in C^\infty (\mathbb {R}^n)$ be a function such that

$$\begin{aligned} \phi (\xi ) = {\left\{ \begin{array}{ll} 0 &  |\xi |\le 1;\\ 1 &  |\xi |\ge 2. \end{array}\right. } \end{aligned}$$

We denote

$$\begin{aligned} A_{\alpha ,\beta }^1f(x) = \int _{\mathbb {R}^n} \int _{\mathbb {R}^n} e^{i(x - y)\xi } (1 - \phi (\xi ))\chi (x) \xi ^\alpha \frac{\mu (x, \xi /|\xi |) D_y^\beta \nu (y, \xi /|\xi |)}{|\xi |^{n-1}} \chi (y) f(y) \text {d}y \text {d}\xi \end{aligned}$$

and

$$\begin{aligned} A_{\alpha ,\beta }^2f(x) = \int _{\mathbb {R}^n} \int _{\mathbb {R}^n} e^{i(x - y)\xi } \phi (\xi ) \chi (x) \xi ^\alpha \frac{\mu (x, \xi /|\xi |) D_y^\beta \nu (y, \xi /|\xi |)}{|\xi |^{n-1}} \chi (y) f(y) \textrm{d}y \textrm{d}\xi . \end{aligned}$$

Then, the last inequality implies

54 $$\begin{aligned} |Af(x)| \le C_\Omega \sum _{\alpha + \beta = \gamma } \left( |A_{\alpha ,\beta }^1f(x)| + |A_{\alpha ,\beta }^2f(x)|\right) . \end{aligned}$$

Let us estimate the $L^2$-norm of $A_{\alpha ,\beta }^1f$. The kernel of $A_{\alpha ,\beta }^1$ is given by

$$\begin{aligned} K_{\alpha ,\beta }(x,y) = \int _{\mathbb {R}^n} e^{i(x - y)\xi } (1 - \phi (\xi )) \xi ^\alpha \frac{\chi (x)\chi (y)\mu (x, \xi /|\xi |) D_y^\beta \nu (y, \xi /|\xi |)}{|\xi |^{n-1}} \text {d}\xi . \end{aligned}$$

We estimate

$$\begin{aligned}&\sup _{y\in \mathbb {R}^n} \int _{\mathbb {R}^n} |K_{\alpha ,\beta }(x,y)| \text {d}x\le \Vert \chi \Vert _{L^\infty (\mathbb {R}^n)}\\  &\quad \int _{\mathbb {R}^n} \xi ^\alpha \frac{(1 - \phi (\xi ))}{|\xi |^{n-1}} \text {d}\xi \sup _{\omega \in \mathbb {S}^{n-1}} \Vert \mu (\cdot ,\omega )\Vert _{L^\infty (\mathbb {R}^n)} \sup _{\omega \in \mathbb {S}^{n-1}} \Vert \nu (\cdot ,\omega )\Vert _{C^{|\beta |}(\mathbb {R}^n)}. \end{aligned}$$

Hence,

$$\begin{aligned} \sup _{y\in \mathbb {R}^n} \int _{\mathbb {R}^n} |K_{\alpha ,\beta }(x,y)| \textrm{d}x = C_\Omega \sup _{\omega \in \mathbb {S}^{n-1}} \Vert \mu (\cdot ,\omega )\Vert _{L^\infty (\mathbb {R}^n)} \sup _{\omega \in \mathbb {S}^{n-1}} \Vert \nu (\cdot ,\omega )\Vert _{C^{|\beta |}(\mathbb {R}^n)}, \end{aligned}$$

and similarly,

$$\begin{aligned} \sup _{x\in \mathbb {R}^n} \int _{\mathbb {R}^n} |K_{\alpha ,\beta }(x,y)| \textrm{d}y = C_\Omega \sup _{\omega \in \mathbb {S}^{n-1}} \Vert \mu (\cdot ,\omega )\Vert _{L^\infty (\mathbb {R}^n)} \sup _{\omega \in \mathbb {S}^{n-1}} \Vert \nu (\cdot ,\omega )\Vert _{C^{|\beta |}(\mathbb {R}^n)}. \end{aligned}$$

Therefore, by Lemma 18.1.12 in [8],

55 $$\begin{aligned} \Vert A_{\alpha ,\beta }^1f\Vert _{L^2(\Omega )} \le C_\Omega \sup _{\omega \in \mathbb {S}^{n-1}} \Vert \mu (\cdot ,\omega )\Vert _{L^\infty (\mathbb {R}^n)} \sup _{\omega \in \mathbb {S}^{n-1}} \Vert \nu (\cdot ,\omega )\Vert _{C^{|\beta |}(\mathbb {R}^n)} \Vert f\Vert _{L^2(\Omega )}. \end{aligned}$$

Next, we estimate the $L^2$-norm of $A_{\alpha ,\beta }^2f$. We denote We denote

$$\begin{aligned} \nu _{\alpha ,\beta }(y,\xi ) = \frac{\xi ^\alpha D_y^\beta \nu (y, \xi /|\xi |)}{|\xi |^{|\alpha |}}\chi (y) \end{aligned}$$

and

$$\begin{aligned} v_{\alpha ,\beta }(\xi ) = \int _{\mathbb {R}^n} e^{-iy\xi } \nu _{\alpha ,\beta }(y,\xi ) f(y) \textrm{d}y, \end{aligned}$$

so that

$$\begin{aligned} A_{\alpha ,\beta }^2f(x)=&\int _{\mathbb {R}^n} e^{ix\xi } \phi (\xi )\frac{\chi (x)\mu (x, \xi /|\xi |)}{|\xi |^{n-1-|\alpha |}} v_{\alpha ,\beta }(\xi ) \text {d}\xi \\=&\int _{\mathbb {R}^n} e^{ix\xi } \phi (\xi ) \frac{\chi (x) \mu (x, \xi /|\xi |)}{|\xi |^{n-1-|\alpha |}} \mathcal {F}[\mathcal {F}^{-1}v_{\alpha ,\beta }](\xi ) \text {d}\xi . \end{aligned}$$

Next, we note that

$$\begin{aligned} \sum _{\tau \le n + 1} \int _{\mathbb {R}^n} \left| D_x^\tau \left( \frac{\chi (x) \phi (\xi )\mu (x, \xi /|\xi |)}{|\xi |^{n-1-|\alpha |}} \right) \right| \textrm{d}x&\le C_\Omega \sup _{\omega \in \mathbb {S}^{n-1} }\Vert \chi (\cdot )\mu (\cdot , \omega )\Vert _{C^{n+1}(\mathbb {R}^n)}\\&\le C_\Omega \sup _{\omega \in \mathbb {S}^{n-1} }\Vert \mu (\cdot , \omega )\Vert _{C^{n+1}(\bar{\Omega })}. \end{aligned}$$

Therefore, Theorem 18.1.11’ in [8] implies that

$$\begin{aligned} \int _{\mathbb {R}^n} \Big | \int _{\mathbb {R}^n} e^{ix\xi } \frac{\chi (x) \phi (\xi )\mu (x, \xi /|\xi |)}{|\xi |^{n-1-|\alpha |}}&\mathcal {F}[\mathcal {F}^{-1}v_{\alpha ,\beta }](\xi ) \text {d}\xi \Big |^2 \text {d}x\\  &\le C_\Omega \sup _{\omega \in \mathbb {S}^{n-1} }\Vert \mu (\cdot , \omega )\Vert _{C^{n+1}(\bar{\Omega })}^2 \Vert \mathcal {F}^{-1}v_{\alpha ,\beta }\Vert _{L^2(\mathbb {R}^{n})}^2 \\  &\le C_\Omega \sup _{\omega \in \mathbb {S}^{n-1} }\Vert \mu (\cdot , \omega )\Vert _{C^{n+1}(\bar{\Omega })}^2 \Vert v_{\alpha ,\beta }\Vert _{L^2(\mathbb {R}^{n})}^2. \end{aligned}$$

Hence,

$$\begin{aligned} \Vert A_{\alpha ,\beta }^2f\Vert _{L^2(\mathbb {R}^{n})} \le C_\Omega \sup _{\omega \in \mathbb {S}^{n-1} }\Vert \mu (\cdot , \omega )\Vert _{C^{n+1}(\bar{\Omega })} \Vert v_{\alpha ,\beta }\Vert _{L^2(\mathbb {R}^{n})}. \end{aligned}$$

By Lemma B.1,

$$\begin{aligned} \Vert v_{\alpha ,\beta }\Vert _{L^2(\mathbb {R}^{n})} \le C_\Omega \sup _{\xi \in \mathbb {R}^n} \Vert \nu _{\alpha ,\beta }(\cdot ,\xi )\Vert _{C^n(\bar{\Omega })} \Vert f\Vert _{L^2(\Omega )}. \end{aligned}$$

We estimate

$$\begin{aligned} \sup _{\xi \in \mathbb {R}^n} \Vert \nu _{\alpha ,\beta }(\cdot ,\xi )\Vert _{C^n(\bar{\Omega })}&= \sup _{\xi \in \mathbb {R}^n} \left\| \frac{\xi ^\alpha D^\beta \nu (\cdot , \xi /|\xi |)}{|\xi |^{|\alpha |}}\chi (\cdot )\right\| _{C^n(\bar{\Omega })}\\  &\le C_\Omega \sup _{\xi \in \mathbb {R}^n} \left\| D^\beta \nu (\cdot , \xi /|\xi |) \right\| _{C^n(\bar{\Omega })}\\&\le C_\Omega \sup _{\xi \in \mathbb {S}^{n-1}} \left\| \nu (\cdot , \xi ) \right\| _{C^{n + |\beta |}(\bar{\Omega })}. \end{aligned}$$

Therefore,

$$\begin{aligned} \Vert A_{\alpha ,\beta }^2f\Vert _{L^2(\mathbb {R}^{n})} \le C_\Omega \sup _{\omega \in \mathbb {S}^{n-1} }\Vert \mu (\cdot , \omega )\Vert _{C^{n+1}(\bar{\Omega })} \sup _{\xi \in \mathbb {S}^{n-1}} \left\| \nu (\cdot , \xi ) \right\| _{C^{n + |\beta |}(\bar{\Omega })} \Vert f\Vert _{L^2(\Omega )}. \end{aligned}$$

Therefore, by (54) and (55), we obtain

$$\begin{aligned} \Vert Af\Vert _{L^2(\mathbb {R}^n)} \le C_\Omega \sup _{\omega \in \mathbb {S}^{n-1} }\Vert \mu (\cdot , \omega )\Vert _{C^{n+1}(\bar{\Omega })} \sup _{\omega \in \mathbb {S}^{n-1}} \left\| \nu (\cdot , \omega ) \right\| _{C^{n -1 + |\gamma |}(\bar{\Omega })} \Vert f\Vert _{L^2(\Omega )}. \end{aligned}$$

Similarly, we estimate $\Vert Bf\Vert _{L^2(\mathbb {R}^n)}$ and conclude that

$$\begin{aligned} \Vert \chi R_\mu ^* R_\nu D^\alpha (\chi f)\Vert _{L^2(\mathbb {R}^n)}\le &   C_\Omega \sup _{\omega \in \mathbb {S}^{n-1} }\Vert \mu (\cdot , \omega )\Vert _{C^{n+1}(\bar{\Omega })} \\    &   \sup _{\omega \in \mathbb {S}^{n-1}} \left\| \nu (\cdot , \omega ) \right\| _{C^{n -1 + |\gamma |}(\bar{\Omega })}\Vert f\Vert _{L^2(\Omega )}. \end{aligned}$$

This completes the proof. $\square$

Now, we prove Theorem B.1.

### Proof of Theorem B.1

Let $f\in C_0^\infty (\Omega )$ and $\Omega \subset \mathbb {R}^n$ compact. We denote $L=(n-1)/2$ and estimate

$$\begin{aligned} \Vert R_\mu f\Vert _{L^2(\mathbb {S}_\omega ^{n-1}; H^{L}(\mathbb {R}_\sigma ))}^2 = \sum _{l=0}^{L} \Vert \partial _p^l R_\mu f\Vert _{L^2(\mathbb {R}\times \mathbb {S}^{n-1})} \le \sum _{l=0}^{2L} |(R_\mu ^*\partial _p^l R_\mu f,f)_{L^2(\mathbb {R}^n)}|. \end{aligned}$$

We note that

$$\begin{aligned} \partial _p^l R_\mu f(p,\omega )&= \partial _p^l \left( \int _{\omega ^\perp } \mu (p\omega + y, \omega ) f(p\omega + y) \textrm{d}y \right) \\&= \sum _{|\alpha | + |\beta |=l} C_{\alpha ,\beta } \int _{\omega ^\perp } D^\alpha f(p\omega + y) D^\beta \mu (p\omega + y, \omega ) \omega ^{\alpha +\beta } \textrm{d}y. \end{aligned}$$

Let us set

$$\begin{aligned} \mu _{\alpha ,\beta }(x,\omega ) = \omega ^{\alpha +\beta } D^\beta \mu (x,\omega ) \end{aligned}$$

so that, the previous equality gives

$$\begin{aligned} \partial _p^l R_\mu = \sum _{|\alpha | + |\beta | = l} C_{\alpha ,\beta } R_{\mu _{\alpha ,\beta }} D^\alpha \quad \text {and} \quad R_\mu ^*\partial _p^l R_\mu = \sum _{|\alpha | + |\beta | = l} C_{\alpha ,\beta } R_\mu ^* R_{\mu _{\alpha ,\beta }} D^\alpha . \end{aligned}$$

By Lemma B.2, we estimate

$$\begin{aligned}&|(R_\mu ^* R_{\mu _{\alpha ,\beta }} D^\alpha f, f)_{L^2(\Omega )}|\\&\quad \le C_\Omega \sup _{\omega \in \mathbb {S}^{n-1}} \Vert \mu (\cdot ,\omega )\Vert _{C^{n+1}(\bar{\Omega })} \sup _{\omega \in \mathbb {S}^{n-1}} \Vert D^{\beta }\mu (\cdot ,\omega )\Vert _{C^{n - 1 + |\alpha |}(\bar{\Omega })} \Vert f\Vert _{L^2(\Omega )}^2\\&\quad \le C_\Omega \sup _{\omega \in \mathbb {S}^{n-1}} \Vert \mu (\cdot ,\omega )\Vert _{C^{2n - 2}(\bar{\Omega })}^2 \Vert f\Vert _{L^2(\Omega )}^2. \end{aligned}$$

This completes the proof. $\square$
